# Prevention of Radiodermatitis With Topical Chinese Herbal Medicine: A Systematic Review and Meta-Analysis

**DOI:** 10.3389/fphar.2022.819733

**Published:** 2022-06-22

**Authors:** Hui-Bo Yu, Bao-Jin Han, Hui-Juan Cao

**Affiliations:** Beijing University of Chinese Medicine, Beijing, China

**Keywords:** radiodermatitis, topical Chinese herbal medicine, systematic review, meta-analysis, radiotherapy

## Abstract

**Objectives:** Topical Chinese herbal medicine (TCHM) is widely used to prevent radiodermatitis in patients who receive radiation therapy in China. However, evidence regarding its efficacy remains limited. The purpose of the review is to evaluate the effects of TCHM in preventing radiodermatitis.

**Methods:** The protocol of this review was registered in PROSPERO (CRD42020220620). Relevant clinical trials were identified (from January 1, 2010, to April 24, 2022) through 11 electronic databases, including PubMed, SpringerLink, Proquest, the Cochrane Central Register of Controlled Trials, Scopus, the ProQuest Dissertation & Theses Global, PsycINFO, Applied Social Sciences Index and Abstracts, the Chinese National Knowledge Infrastructure Databases, Wangfang Data Knowledge Service Platform, and the Chongqing VIP Chinese Science and Technology Periodical Database. The quality of the included trials was assessed through a risk of bias assessment using Version 2 of the Cochrane risk-of-bias tool (RoB 2.0). We included RCTs that compared TCHM single used or as adjunctive treatment with routine drugs, conventional therapy, or placebo for cancer patients who are about to start radiation therapy and do not possess any type of dermatitis or skin lesions at that time. Primary outcomes of interest were the incidence of radiodermatitis and the grade of radiodermatitis according to the RTOG (Radiation Therapy Oncology Group). Secondary outcomes included the recovery time of skin and mucosa, the occurrence time of radiodermatitis, the radiation dose, quality of life, and adverse events. Data were summarized using risk ratio (RR) calculations and 95% confidence intervals (CI) for binary outcomes or mean difference (MD) with 95% CI for continuous outcomes. Certainty of the evidence was assessed according to the GRADE criteria.

**Results:** In this review, 38 randomized controlled trials (RCTs) were included. Risk of bias assessment through RoB 2.0 showed that two studies were rated as low risk, two studies were rated as high risk, and the rest were rated as having some concerns. Compared with routine drugs, TCHM may have an advantage in reducing RTOG grading (RR = 0.46, 95%CI 0.35–0.60), decreasing the recovery time of radiodermatitis (MD = −2.35, 95%CI 3.58 to −1.12 days), delaying the occurrence of radiodermatitis (MD = 2.36, 95%CI 1.74–2.98), and improving the quality of life of patients (RR = 1.46, 95%CI 1.03–2.06). Compared with conventional therapy, TCHM may also have an advantage in decreasing the grade of RTOG (RR = 0.28, 95%CI 0.21–0.38).

**Conclusion:** Current low evidence revealed that TCHM may have better efficacy in the prevention of radiodermatitis; however, more high-quality RCTs are still warranted to testify this conclusion.

**Systematic Review Registration:** (https://www.crd.york.ac.uk/prospero/display_record.php?ID=CRD42020220620), identifier (PROSPERO 2020 CRD42020220620).

## 1 Introduction

Radiodermatitis (RD) is one of the most common side effects of radiation therapy for cancer (Ashack et al., 2020; Behroozian et al., 2021; Yao and Cheng, 2021). At present, about 95% of patients who receive radiation therapy will develop RD (Rosenthal et al., 2019). Patients with breast cancer, head and neck cancer, lung cancer, or sarcoma are more likely to suffer from RD (Fuzissaki et al., 2019; Tinkle et al., 2021; Yokota et al., 2021; Louie et al., 2022). Erythema is the predominant sign among RD patients, while 42 and 15% of patients developed RT-induced grade 3 and 4 skin toxicities characterized by desquamation, ulceration, and pigment changes (Hu et al., 2018; Bontempo et al., 2021). The consequences of RD can be serious, including dose reduction or discontinuation of therapy, delayed treatment, diminished esthetic appeal, and significantly impaired quality of life.

The main risk factors for RD are both treatment and patient-related, they may relate to the parts of the body that received radiation therapy (Xie et al., 2021), coexisting diseases (such as diseases related to DNA repair defects), obesity, advanced age, female, long-term sun exposure, smoking (Hymes et al., 2006; Meyer et al., 2012; Bray et al., 2016), total radiation dose, segmented metering, and exposed volume and surface area (Kole et al., 2017; Liu et al., 2020). It has been reported that traditional chemotherapy or EGFR inhibitors such as anthracyclines or taxanes will increase the risk of severe RD (Tejwani et al., 2009).

General management of RD includes daily preventive measures and topical or systemic pharmaceutical treatment. Daily preventive measures include keeping the radiation therapy area clean and dry, washing with warm water and mild soap, avoiding skin irritation, wearing loose clothing, preventing abrasions, etc. (Finkelstein et al., 2022; Yang et al., 2020). Recommendations for use of topical skin agents including glucocorticoids, Vaseline ointment, olive oil, ascorbic acid, sulfadiazine, etc. (Finkelstein et al., 2022; Lee et al., 2016; Menon et al., 2021). Recommendations for systemic drugs include protease (Gujral et al., 2001), Pentoxifylline (Aygenc et al., 2004), antioxidants (Lin et al., 2006), etc. Previous published systematic reviews (Miller et al., 2011; Chan et al., 2014; Hindley et al., 2014; Ulff et al., 2017) showed that cleaning in daily care can reduce itching, redness, and peeling, but does not reduce the overall risk of radiation dermatitis, regular topical use of corticosteroids can reduce the incidence of severe dermatitis during radiation therapy and within a few weeks after radiation therapy. However, the use of glucocorticoids during radiation therapy did not reduce the incidence of RD (Chan et al., 2014) and there is no evidence to support the standard approach for the prevention and treatment of RD.

Meanwhile, several studies have shown that topical Chinese herbal medicine (TCHM) has certain advantages in the prevention of RD, it may reduce the incidence of grade 3 and 4 RD according to RTOG (Wang et al., 2016; Wu, 2019), shorten the course of the disease, and delay the occurrence of dermatitis (Wang et al., 2014). Some traditional Chinese herbals and compound prescriptions such as aloe, moist burn ointment, and Kangfuxin liquid (dry insect extract of *Periplaneta americana*) have been proved to promote the proliferation and renewal of granulation tissue and improve the blood circulation of the wound and its surroundings to promote the repair and regeneration of wound skin (Wu et al., 2010; Wei, 2012; Zhu et al., 2013). Curcumin in turmeric has the effect of scavenging free radicals, increasing the activity of oxidase, anti-inflammation, and anti-infection (Huang et al., 2016). Compound ulcer oil can inhibit the production and release of interleukin-6 (IL-6), tumor necrosis factor-α (TNF-α), IL-1, and IL-8, and promote the production of epidermal growth factor (EGF), thus delaying the occurrence of skin inflammation and improving the speed of recovery (Zhao et al., 2016).

RD may lead to delays in treatment, diminished cosmesis, a decline in quality of life, and functional deficits. Although it confuses clinicians and patients, there is no gold standard in the prevention and management of this condition, and many interventions are based on provider experience, anecdotal evidence, or poorly powered studies (Leventhal and Young, 2017). TCHM is widely used in China, and many clinical studies have reported that it has preventive and treatment effects on RD. To some extent, prevention is more valuable than treatment (Martin et al., 2020). Therefore, we conducted this systematic review of TCHM for RD prevention to obtain evidence.

## 2 Methods

### 2.1 Study Design

Protocol of this review was registered in PROSPERO as Huibo Yu, Baojin Han, and Huijuan Cao. Prevention of radiodermatitis with topical medication of Chinese herbal medicine: a systematic review of randomized controlled trials. PROSPERO 2020 CRD42020220620. Available from: https://www.crd.york.ac.uk/prospero/display_record.php?ID=CRD42020220620. We conducted the systematic review in strict accordance with the predefined protocol.

The reporting of this systematic review adheres to the Preferred Reporting Items for Systematic Reviews and Meta-Analysis (PRISMA) Checklist.

### 2.2 Eligibility Criteria

In Chinese or English, studies were included when the following criteria were met: 1) Patient: *Cancer* patients, of any age, who is about to start radiation therapy and do not possess any type of dermatitis or skin lesions before the radiotherapy. 2) Intervention: TCHM, including herbal patent, preparation produced in hospitals, herbal lotion, etc. 3) Comparators: Controls include routine drugs (such as Recombinant Human Epidermal Growth Factor Derivative for External Use, Trolamine Cream, Hydrocortisone Butyrate Cream), conventional therapy (proper rest, routine radiodermatitis missionary, vitamin, and other energy), placebo or no treatment. 4) Outcomes: primary outcomes included incidence rate of radiodermatitis and RTOG (Radiation Therapy Oncology Group) Grading criteria. Secondary outcomes included recovery time of skin mucosa, time of incidence of radiodermatitis, exposure dose in the incidence of radiodermatitis, quality of life (e.g., Karnofsky Performance Status, KPS), and adverse events. 5) Study type: Randomized controlled trials (RCTs). Literature that is unable to obtain the full text or the analyzable data was excluded.

### 2.3 Exclusion Criteria

1) Insufficient data. 2) Irregular outcome evaluation criteria.

### 2.4 Literature Searching

PubMed, SpringerLink, Proquest, the Cochrane Central Register of Controlled Trials (CENTRAL), Scopus, the ProQuest Dissertation & Theses Global (PQDT), PsycINFO, Applied Social Sciences Index and Abstracts (ASSIA), the Chinese National Knowledge Infrastructure Databases (CNKI), Wangfang Data Knowledge Service Platform, and the Chongqing VIP Chinese Science and Technology Periodical Database (VIP) were searched from 1 January 2010 to 24 April 2022, in Chinese and English. The searching terms of retrieval strategy were different based on each database, which included “radio dermatitis,” or “radiation reaction,” or “radioactive lesions,” or “radio epithelitis” combined with “Chinese medicine,” or “herbal medicine,” or “herbs,” or “TCM,” and combined with “random.” We searched for additional trials by reviewing the reference lists of studies related to the prevention of RD with TCHM. All studies were independently searched by two reviewers (HBY and BJH). Any disagreement was resolved through the third reviewer (HJC).

### 2.5 Study Screening and Data

Studies using the search strategy were retrieved and the full text of these potentially eligible studies was retrieved and independently assessed for eligibility by two reviewers (HBY and BJH). Any disagreement was resolved through discussion with the third reviewer (HJC).

Extracted information included: General information (document number, title, first author, year(s), location (city, country), source, etc.), methodological related information (study design type, random scheme concealment, random allocation method, randomization blind method, statistical analyst blinded, loss of follow-up, baseline comparability, and selective reporting), participants information (diagnostic criteria, inclusion criteria, source, sample size, age, gender, types of carcinoma, and site of radiation exposure), intervention information (types of experimental group interventions, types of control group interventions, and treatment duration), and outcome relevant information (incidence rate of radiodermatitis, RTOG Grading criteria, exposure dose in the incidence of radiodermatitis, the recovery time of skin mucosa, time of incidence of radiodermatitis, and quality of life). We intended to contact the original authors of the included trials to acquire the missing information if needed; however, we found no key information that might affect our judgment of inclusion was missing.

### 2.6 Methodological Quality Assessment

Two reviewers (HBY and BJH) independently evaluated the included studies, and any disagreement arising from this process was resolved by a third reviewer (HJC). Risk of bias assessment Version 2 of the Cochrane risk-of-bias tool (RoB 2.0) was used to assess the quality of included studies, in which five domains were assessed: randomization process, deviations from the intended intervention, missing outcome data, measurement of the outcome, and selection of the reported result. Within each domain, a series of questions were designed to elicit information about trial features associated with bias risk, making the assessment more accurate. Overall bias was considered as “low-risk” if the study was classified as low-risk in all domains, “some concerns” if there was at least 1 domain rated as having some concerns, and “high-risk” if there was at least 1 domain rated as high-risk or several domains rated as having some concerns that could affect the validity of the results.

### 2.7 Data Analysis

All statistical analyses were performed using RevMan 5.4 (The Cochrane Collaboration) software. Data were summarized using risk ratio (RR) calculations and 95% confidence intervals (CI) for binary outcomes or mean difference (MD) with 95% CI for continuous outcomes.

Intention to treat analysis was not employed to deal with the missing data, since almost all the included trials reported no missing data during the data collection and analysis. Statistical heterogeneity among included trials was evaluated by the *I* ^*2*^ test. Meta-analysis was conducted if there is no significant clinical and statistical heterogeneity (*I* ^*2*^ < 75%) among included trials. Considering the potential clinical heterogeneity among trials caused by the effect of different prescriptions, we used random-effects model to pool the data to ensure the rationality of the results. Pooling analysis was not used if there is significant statistical heterogeneity (*I* ^*2*^ ≥ 75%) among trials. The funnel plot was used to explore the potential publication bias if there are ten or more trials in the meta-analysis.

Subgroup analysis was performed when there was significant heterogeneity (*I* ^*2*^ > 25%) in the results. The subgroups were classified by the effects of different radiation therapy dosages, different radiation dermatitis and mucositis grading, different western drugs, different types of carcinoma, or different dosage forms of the TCHM on the results. Sensitivity analysis was conducted to challenge the robustness of the primary analysis, for trials with or without a high-risk of bias, and for meta-analyses conducted using the fixed-effect model or the random-effect model.

We used the Grading of Recommendations Assessment Development and Evaluation Criteria (GRADE) to assess the certainty of the evidence for outcomes with meta-analysis. By assessing the risk of bias, inconsistency of trials, indirectness of evidence, imprecision of results, and publication bias, we determined whether to downgrade the evidence. The evidence was assessed on four levels: high, moderate, low, or very low.

## 3 Results

### 3.1 Searching of the Literature

**FIGURE 1:**
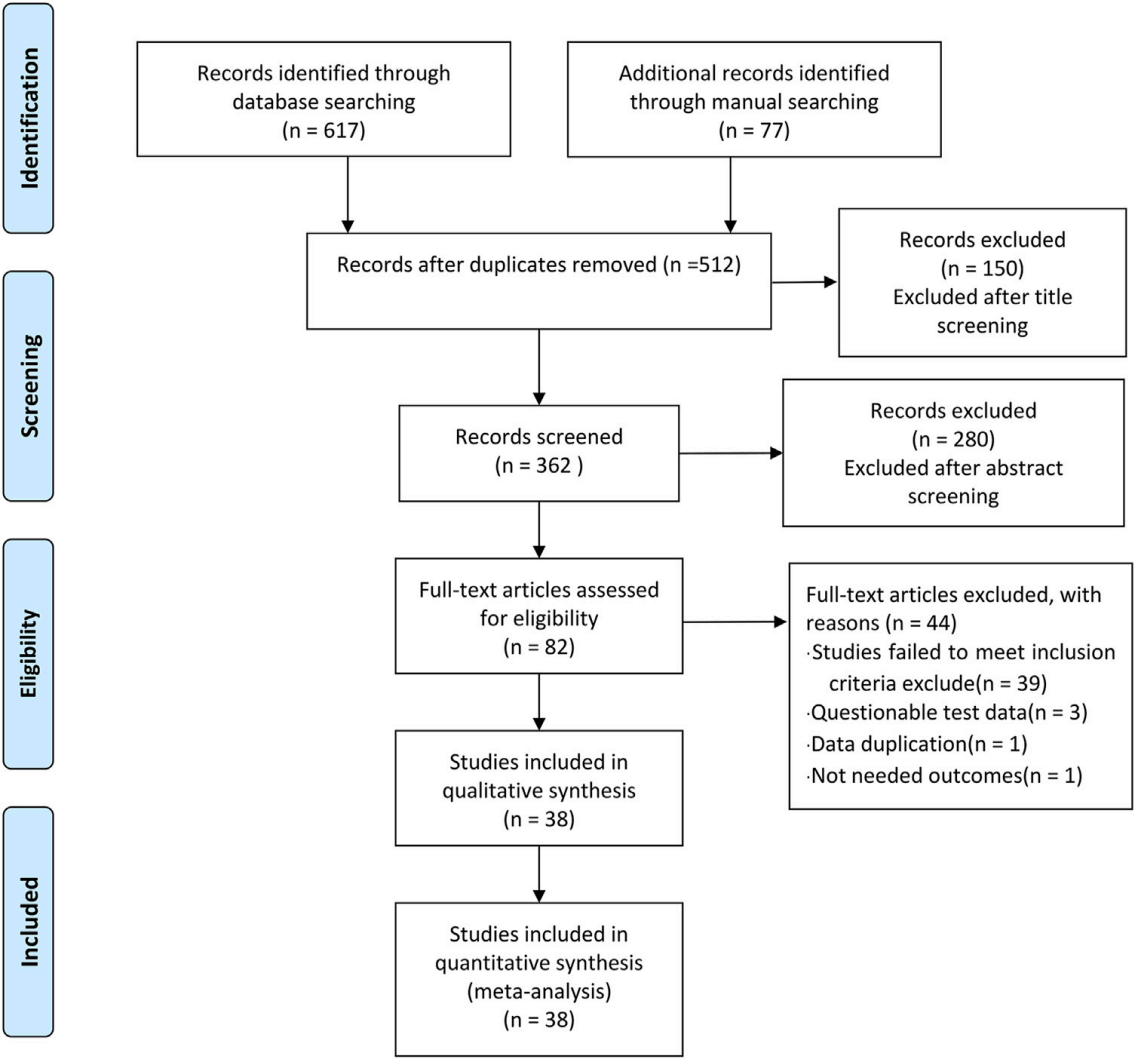
Study flow chart.

In phase 1 of study selection, 674 articles were identified across 11 electronic databases and through manual searching. After the duplicated articles were removed, 512 articles remained. A thorough screening of the title was completed, and 150 articles were excluded. Two hundred and eighty articles were further excluded through the abstracts screening. In phase 2, 82 articles remained for full-text screening. This process led to the exclusion of 44 articles. In total, 38 articles were selected for data extraction and qualitative synthesis. The specific process is shown in Figure 1.

### 3.2 Characteristics of the Included Studies

**FIGURE 2:**
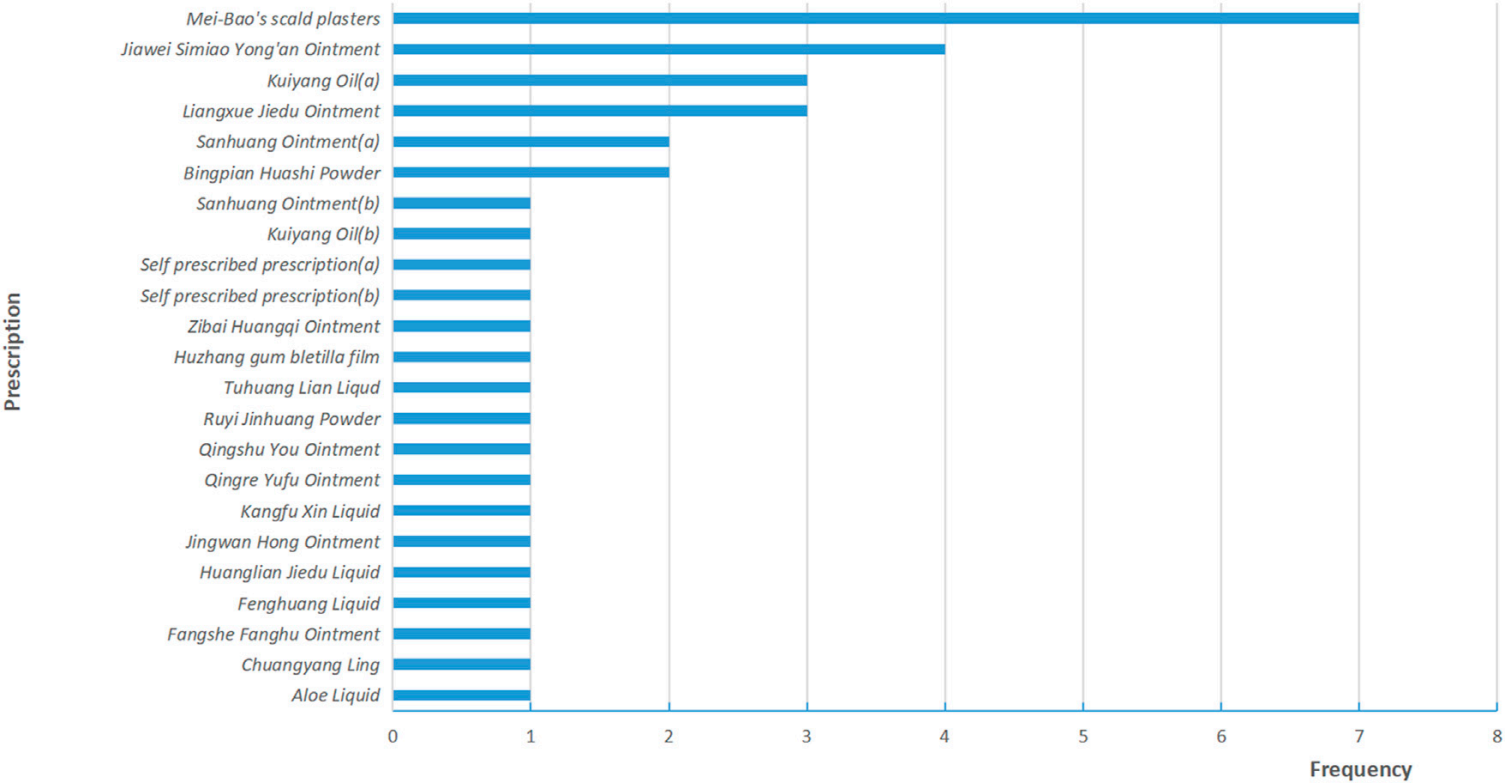
Frequency distribution of prescription.

**FIGURE 3:**
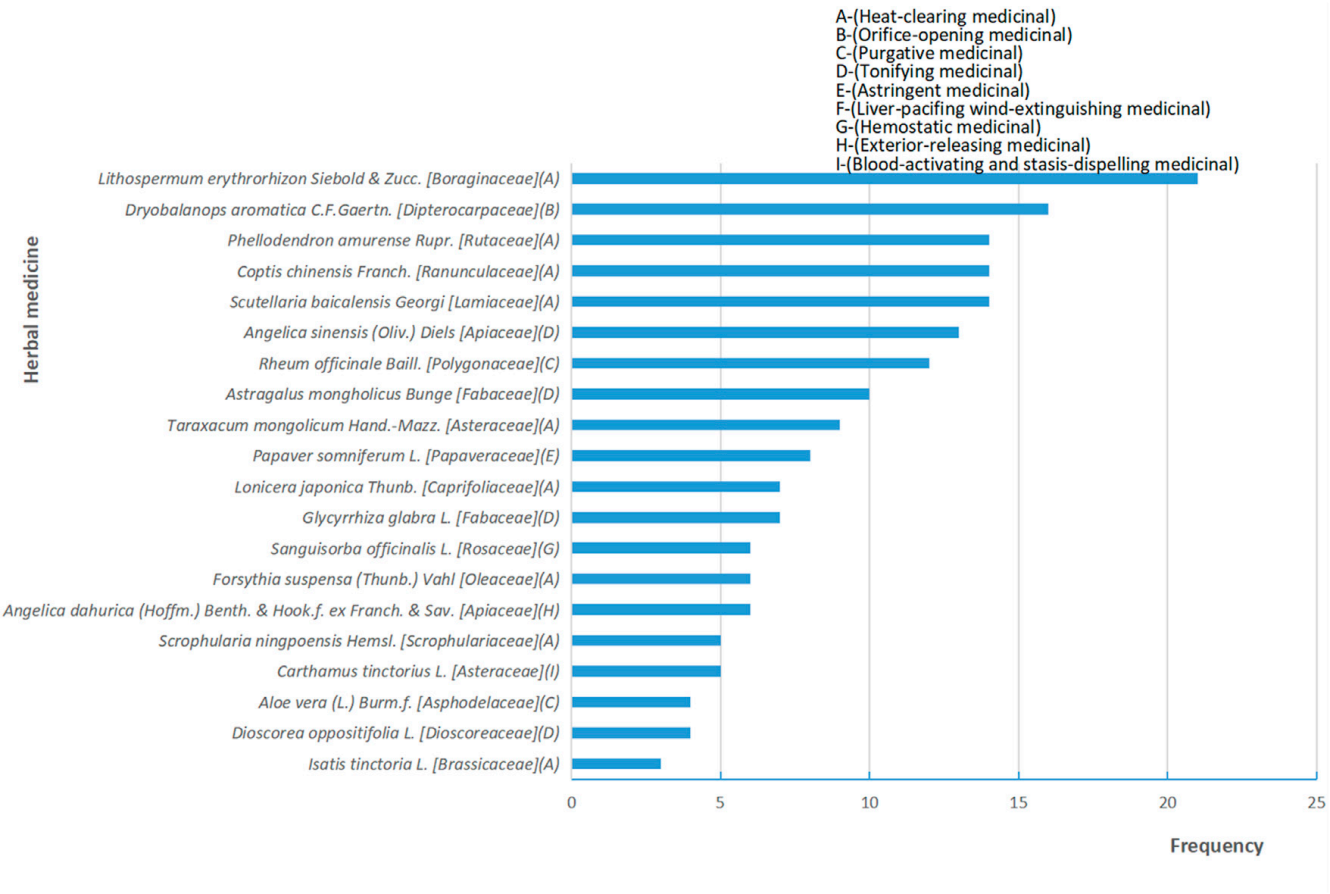
Frequency distribution and functional classification of herbal medicine.

A total of 38 RCTs (Li et al., 2010; Luo and Lan, 2010; Yu et al., 2010; Zhang and Xue, 2010; Bian et al., 2011; Guo and Shi, 2011; Li et al., 2011; Wang C. et al., 2011; Wang J. et al., 2011; Li et al., 2013; Li and Liu, 2013; Lin et al., 2013; Niu, 2013; Liang and Huang, 2014; Liu et al., 2014; Wang et al., 2014; Xu et al., 2014; Yang and Guan, 2014; Zhang, 2014; Zu et al., 2014; Mao, 2015; Wang et al., 2015; Zhang, 2015; Wang et al., 2016; Yang, 2016; Zhao et al., 2016; Tang et al., 2017; Yuan et al., 2017; Geara et al., 2018; Wang et al., 2018; Dong, 2019; Liao et al., 2019; Wang et al., 2019; Wu, 2019; Song et al., 2020; Lei et al., 2021; Liu, 2021; Zhao et al., 2022) and 3,439 patients were included in this review, 1724 patients were treated by TCHM. In TCHM group, ointment was used in 21 studies (Zhang and Xue, 2010; Guo and Shi, 2011; Li et al., 2011; Wang C. et al., 2011; Li et al., 2013; Niu, 2013; Xu et al., 2014; Yang and Guan, 2014; Zhang, 2014; Zu et al., 2014; Mao, 2015; Zhang, 2015; Yuan et al., 2017; Geara et al., 2018; Dong, 2019; Wang et al., 2019; Wu, 2019; Song et al., 2020; Lei et al., 2021; Liu, 2021; Zhao et al., 2022), oil was used in 8 studies (Yu et al., 2010; Bian et al., 2011; Liang and Huang, 2014; Liu et al., 2014; Wang et al., 2015; Wang et al., 2016; Yang, 2016; Zhao et al., 2016), solution was used in 5 studies (Wang J. et al., 2011; Lin et al., 2013; Tang et al., 2017; Wang et al., 2018; Liao et al., 2019), powder was used in 3 studies (Li et al., 2010; Luo and Lan, 2010; Li and Liu, 2013), and TCM drug films were used in 1 study. The general principle of treatment included clearing heat-toxin, supplemented by replenishing qi and nourishing yin, activating blood, resolving stasis, and relieving pain. A total of 23 prescriptions were used in all experiments, involving 53 kinds of herbal medicine. The herbal medicine repetition rate in all prescriptions was 49.1%. All the prescriptions have the effect of clearing the heat-toxin. The specific prescription, herbal and dosage, treatment principle, and general medicine are shown in Table 1. The frequency of prescriptions is shown in Figure 2, and the frequency of the top 20 herbs is shown in Figure 3. The controls included routine drugs and conventional therapy. There were 18 RCTs, including 794 patients who employed routine drugs as control. Triethanolamine was the most commonly used drug. Other 20 RCTs, including 921 patients, used conventional therapy in the control group. The main cancer types were breast cancer and head and neck cancer, and the radiation dose levels ranged from 45 to 80 Gy. All the studies reported the incidence of RD, 37 studies reported the RTOG grading of RD, 6 studies reported the occurrence time of RD, 7 studies reported the recovery time of RD, none of the trials reported the exposure dose for RD, 6 studies reported the patients’ change in the quality of life, and adverse events were reported in 13 studies. The basic characteristics of the included literature are shown in Table 1.

**TABLE 1:** Characteristics of studies on prevention of radiodermatitis with TCHM.

Study ID		Simple size	*Cancer* type	Age (yrs, X±SD)	Dosage (Gy)	Course of treatment	Intervention	Control	Outcome
**1. TCHM VS. conventional therapy**									
BianJL2011		T = 60	BC	T:26–55	50	Entire Radiation Cycle	Self-prescribed prescription(a)	Conventional therapy	12
		C = 60		C:30–57					
DongQ2019		T = 15	BC	T:37.52 ± 2.10	51	Entire Radiation Cycle+2-3w	Mei-Bao’s scald plasters	Conventional therapy	123
		C = 15		C:37.45 ± 2.11					
GuoYH2011		T = 23	NPC	24–62	Not Mentioned	Entire Radiation Cycle	Mei-Bao’s scald plasters	Conventional therapy	12
		C = 23							
LeiL2021		T = 38	BC	30–70	50	Entire Radiation Cycle	Zibai Huangqi Ointment	Conventional therapy	12,345
		C = 38							
LiangJ2014		T = 79	LCA, BC, HCC	T: Median49	50–76	Entire Radiation Cycle	Zicao Diyu Oil	C1: Mucopolysaccharide Polysulfate Cream	1
		C1 = 69		C1:Median51				C2: Conventional therapy	
		C2 = 73		C2:Median54					
LiJ2011		T = 50	BC	T:40–65	Not Mentioned	Entire Radiation Cycle	Liangxue Jiedu Ointment	Conventional therapy	12
		C = 50		C:42–63					
LiS2013		T = 63	BC	Not Mentioned	Not Mentioned	5w	Tuhuang Lian Liqud	Conventional therapy	12
		C = 63							
LiuXJ2021		T = 20	BC, LCA, gastrointestinal carcinomas, genitourinary system carcinomas	T:34–84 Median64	T:43.9 ± 10.41	Entire Radiation Cycle+2w	Jiawei Simiao Yong’an Ointment	Conventional therapy	1,236
		C = 20		C:47–87 Median64.5	C:43.7 ± 10.29				
LiXH2010		T = 30	NPC	24∼69	66–70	Entire Radiation Cycle	Bingpian Huashi Powder	Conventional therapy	12
		C = 30							
LuoAJ2010		T = 50	LCA, laryngocarcinoma, Lymphoma	T:43 ± 5	Not Mentioned	Entire Radiation Cycle	Bingpian Huashi Powder	Conventional therapy	12
		C = 50		C:44 ± 4					
MaoWP2015		T = 20	LCA, ESCA, head-neck tumors, BC, CRC, bladder cancer	T:62.40 ± 8.80	50–70	Entire Radiation Cycle	Jiawei Simiao Yong’an Ointment	Conventional therapy	1,236
		C = 20		C:62.50 ± 11.29					
SongFL2019		T = 20	NPC	T:58.20 ± 12.73	20–70	Entire Radiation Cycle	Jiawei Simiao Yong’an Ointment	Conventional therapy	12
		C = 20		C:56.85 ± 12.47					
WangCZ2011		T = 49	BC	T:23–69	50	Entire Radiation Cycle	Liangxue Jiedu Ointment	Conventional therapy	12
		C = 47		C:23–70					
WangYC2019		T = 90	UCC	T:51.85 ± 7.63	10–60	Entire Radiation Cycle+2w	Mei-Bao’s scald plasters	Conventional therapy	124
		C = 90		C:52.08 ± 8.16					
WangYH2014		T = 53	BC, ESCA, NPC, TCS, oral cancer, Malignant lymphoma of the neck	28–80	45–50	35d	Huzhang gum bletilla film	Conventional therapy	12,345
		C = 49							
YangXY2014		T = 56	BC	24–57	Not Mentioned	Entire Radiation Cycle	Jingwan Hong Ointment	C1:Propole Cream	12
		C = 56						C2:Conventional therapy	
ZhangJK2015		T = 140	NPC, BC, ESCA, LCA	29–76	48–70	Entire Radiation Cycle+1d	Liangxue Jiedu Ointment	Conventional therapy	123
		C = 140							
ZhangSS2014		T = 36	BC	32–65	50	6w+2–3W	Mei-Bao’s scald plasters	Conventional therapy	12
		C = 36							
ZhangWW2010		T = 30	BC	32–65	50	5w	Mei-Bao’s scald plasters	Conventional therapy	12
		C = 30							
ZuGH2014		T = 13	BC	26–65	50–66	5w+2∼3w	Fangshe Fanghu Ointment	C1:Piji Ling Cream	12
		C1 = 13						C2:Trolamine Cream	
		C2 = 13						C3:Conventional therapy	
		C3 = 13							
**2. TCHM VS. routine drugs**									
Fady BG2018		T = 78	BC	T:51.73 ± 11.23	<50	Entire Radiation Cycle+2w	Mei-Bao’s scald plasters	Trolamine Cream	12
		C = 70		C:50.19 ± 12.57					
LiaoDZ2019		T = 45	NPC, laryngocarcinoma, THCA, Maxillary Sinus *Cancer*, NK/T Cell Lymphoma, Oral *Cancer*	T:45.5 ± 9.63	66–70	5∼7w	Huanglian Jiedu Liquid	Recombinant Human Epidermal Growth Factor Derivative For External Use, Liquid	1,235
		C = 45		C:47.5 ± 7.52					
LiJH2013		T = 40	LCA, BC, Rectal Carcinoma, Lymphoma	T:56.8 ± 3.7	50–80	Not Mentioned	Ruyi Jinhuang Powder	Recombinant Human Epidermal Growth Factor Derivative For External Use, Liquid	12
		C = 40		C:51.6 ± 6.2					
LiuFF2014		T = 34	NPC, laryngocarcinoma, Tongue *Cancer*, Paranasal sinus carcinoma	T:42.0 ± 3.6	60–70	Entire Radiation Cycle	Self-prescribed prescription(b)	Superoxide Dismutase, liquid	12
		C = 34		C:41.3 ± 3.5					
LiXJ2013		T = 30	BC	40–60	45–50	35d	Sanhuang Ointment(a)	Trolamine Cream	1,245
		C = 30							
NiuLY2013		T = 32	NPC	16–78	50–74	Entire Radiation Cycle	Mei-Bao’s scald plasters	Trolamine Cream	12
		C = 31							
TangHJ2017		T = 15	BC	53.89 ± 3.27	50–70	5-7w	Fenghuang Liquid	Trolamine Cream	12
		C = 15							
WangHY2018		T = 36	BC	26–69	50–60	5-6w+1w	Aloe Liquid	Trolamine Cream	12
		C = 36							
WangJ2016		T = 75	Tonsil carcinoma, Laryngocarcinoma, NPC	T: 43.7 ± 6.1	Not Mentioned	Not Mentioned	Kuiyang Oil(a)	Trolamine Cream	124
		C = 75		C: 43.5 ± 5.9					
WangJG2011		T = 60	BC	T:Median48	50–60	5-6w	Kangfu Xin Liquid	Mucopolysaccharide Polysulfate Cream	12
		C = 60		C:Median49					
WangXP2015		T = 38	NPC	Median45	66–76	6w	Kuiyang Oil(a)	Trolamine Cream	123
		C = 36							
WuH2019		T = 60	ESCA	Median55	59.4–60	6-7w+2w	Qingshu You Ointment	Vaseline Ointment	1,236
		C = 60							
XuY2014		T = 40	BC	T:63.2 ± 1.8	50	5W	Sanhuang Ointment(b)	Magnesium Sulfate Ointment	123
		C = 40		C:63.6 ± 1.9					
YangWB2016		T = 30	LCA, BC, ESCA, NPC, Parotid Carcinoma, GC, UCC	T:59.83 ± 12.05	45–70	Entire Radiation Cycle+1w	Jiawei Simiao Yong’an Ointment	Trolamine Cream	1,236
		C = 30		C:59.93 ± 12.84					
YuanHF2017		T = 63	BC	T:46.35 ± 13.34	50	Entire Radiation Cycle	Chuangyang Ling	Compound Dexamethasone Acetate	126
		C = 63		C:47.93 ± 13.19					
YuZY2010		T = 30	NPC, laryngocarcinoma, Tonsil carcinoma	T:42.14 ± 8.69	50–75	Entire Radiation Cycle	Kuiyang Oil(a)	Trolamine Cream	1,236
		C = 30		C:41.02 ± 10.37					
ZhaoRL2016		T = 40	BC, NPC, Skin *Cancer*	T:46.35 ± 13.34	≥60	Entire Radiation Cycle	Kuiyang Oil(a)	Hydrocortisone Cream	1,245
		C = 40		C:47.93 ± 13.19					
ZhaoSL2022		T = 52	NPC, laryngocarcinoma, melanoma, oral cancer	T:57.58 ± 5.14	55–75	Entire Radiation Cycle+1w	Qingre Yufu Ointment	Trolamine Cream	12,345
		C = 52		C:58.05 ± 5.23					

TCHM: topical Chinese herbal medicine, T: treatment, C: control, ‘X: average, SD: standard deviation, BC: breast cancer, NPC: nasopharynx cancer, LCA: lung cancer, HCC: hepatocellular carcinoma, ESCA: esophageal carcinoma, GC: gastric cancer, TCS: thyroid carcinosarcoma, THCA: thyroid carcinoma, UCC: uterine cervix cancer, CRC: colorectal cancer. 1. Incidence of Dermatitis. 2. RTOG. 3. Adverse Events. 4. Skin and mucosa recovery time. 5. The occurrence time of radiation dermatitis. 6. Quality of Life.

### 3.3 Study Quality Assessment

**FIGURE 4:**
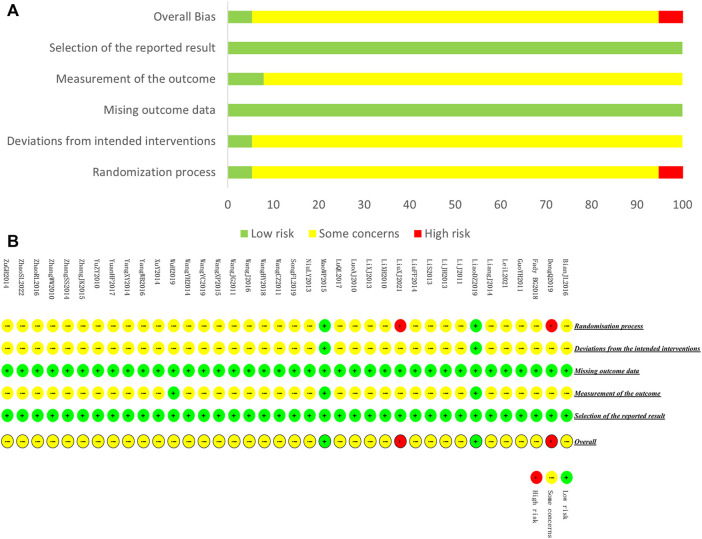
Risk of bias of included studies. **(A)** Risk of bias graph **(B)** Risk of bias summary.

We assessed the risk of bias for 38 included studies. For “Randomization process,” only one study used envelopes to perform allocation concealment (Mao, 2015), and 1 study mentioned double-blind (Liao et al., 2019). These 2 studies were rated as low-risk. Two studies (Dong, 2019; Liu, 2021) were rated high-risk because they generated the random sequence based on the admission order. The rest of the studies were rated with some concerns. For “Deviations from the intended interventions,” only 2 studies were rated as low-risk because the randomization of these studies was reasonable, while the rest of the studies were rated as having some concerns since the absence of blinding may induce the detection bias. For “Missing outcome data,” all the included studies were evaluated as low-risk of bias because of the potential completeness of the data. For “Measurement of the outcome,” only two studies that applied blind method were rated as low-risk. For “Selection of the reported result,” all studies were assessed as having a low-risk of bias because their experimental analysis methods were consistent with the present methods. A summary of the results of the risk of bias assessment for included studies is shown in Figure 4.

### 3.4 Estimate Effects (See Table 2)

#### 3.4.1 Topical Chinese Herbal Medicine vs. Routine Drugs

A total of 18 studies contributed data to this comparison.

##### 3.4.1.1 The Incidence of Radiation Dermatitis/Radiation Therapy Oncology Group Grading

**FIGURE 5:**
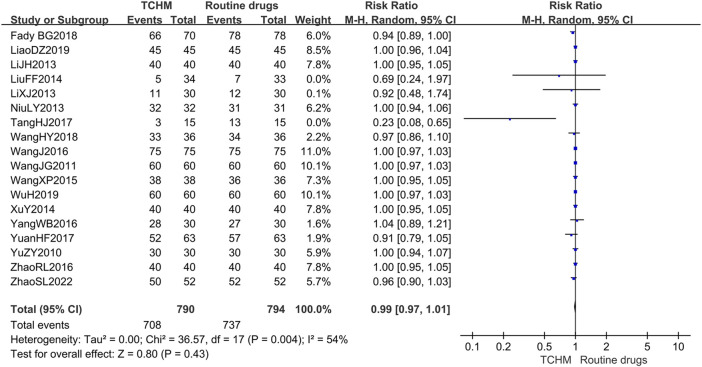
Forest plot and pooled risk ratios for association of incidence rate of radiodermatitis with TCHM and routine drugs.

**FIGURE 6:**
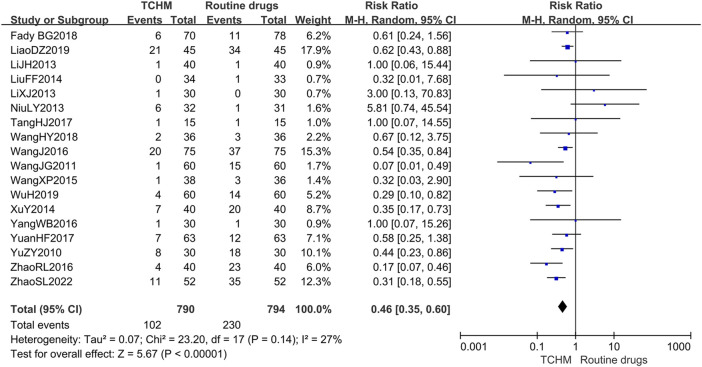
Forest plot and pooled risk ratios for association of RTOG grading criteria with TCHM and routine drugs.

A total of 1,584 patients in 18 studies reported the incidence of RD (Yu et al., 2010; Wang C. et al., 2011; Li et al., 2013; Li and Liu, 2013; Niu, 2013; Liu et al., 2014; Xu et al., 2014; Wang et al., 2015; Wang et al., 2016; Yang, 2016; Zhao et al., 2016; Tang et al., 2017; Yuan et al., 2017; Geara et al., 2018; Wang et al., 2018; Liao et al., 2019; Wu, 2019; Zhao et al., 2022) (shown in Figure 5). Meta-analysis showed that there was no significant difference in the incidence of RD between TCHM and routine drugs (REM, RR = 0.99, 95%CI 0.97 to 1.01, 1,584 participants, *I* ^*2*^ = 54%, *p* = 0.43). The subgroups classified by types of cancer, radiation dose, and dosage form of the TCHM could not explain the source of heterogeneity. We found that heterogeneity was mainly derived from TangHJ 2017. After excluding this study, the heterogeneity dropped to zero. The false-positive result may be caused by the too small sample size of this study (T/C = 15/15). But the meta-analysis also showed that compared with TCHM, routine drugs had an advantage in reducing RTOG grading (REM, RR = 0.46, 95%CI 0.35 to 0.60, 1,584 participants, *I* ^*2*^ = 27%, *p* < 0.00001, see Figure 6). Subgroups classified by types of cancer showed some differences between subgroup (*p* = 0.10, *I* ^*2*^ = 51.6%).

##### 3.4.1.2 Skin and Mucosa Recovery Time

Four studies including 301 patients reported skin and mucosa recovery time (Li et al., 2013; Wang et al., 2016; Zhao et al., 2016; Zhao et al., 2022). Meta-analysis showed that compared with routine drugs, TCHM may reduce the recovery time of RD (REM, MD = −2.35, 95%CI −3.58 to −1.12 days, 301 participants, *I* ^*2*^ = 68%, *p* = 0.0002) (shown in Supplementary Figure S1).

##### 3.4.1.3 The Occurrence Time of Radiation Dermatitis

Four studies including 334 patients reported the occurrence time of RD (Li et al., 2013; Zhao et al., 2016; Liao et al., 2019; Zhao et al., 2022). Meta-analysis showed that TCHM may delay the occurrence of RD compared to routine drugs (REM, MD = 2.36, 95%CI 1.74 to 2.98, 334 participants, *I* ^*2*^ = 0%, *p* < 0.00001) (shown in Supplementary Figure S2).

##### 3.4.1.4 The Radiation Dose

No study mentioned this outcome.

##### 3.4.1.5 Quality of Life

Four studies including 366 patients reported quality of life, of which 180 cases in two studies reported changes in the quality of life before and after treatment (Yang, 2016; Wu, 2019). Due to huge heterogeneity, we were unable to conduct a meta-analysis; however, results from each trial showed no difference in this outcome between groups.

Meta-analysis of another two studies (Yu et al., 2010; Yuan et al., 2017) showed that TCHM had an advantage in improving the quality of life in patients with RD compared with routine drugs (REM, RR = 1.46, 95%CI 1.03 to 2.06, 186 participants, *I* ^*2*^ = 0%, *p* = 0.03) (shown in Supplementary Figure S3).

##### 3.4.1.6 Adverse Events

Seven studies mentioned the adverse events (Yu et al., 2010; Xu et al., 2014; Wang et al., 2015; Yang, 2016; Liao et al., 2019; Wu, 2019; Zhao et al., 2022), and six of them reported no adverse events. The remaining study reported that one patient in the treatment group had a rash in the smeared area and two patients in the control group had pruritus in the smeared area (Wang et al., 2015).

#### 3.4.2 Topical Chinese Herbal Medicine vs. Conventional Therapy

##### 3.4.2.1 The Incidence of Radiation Dermatitis

A total of 1855 patients in 20 studies reported the incidence of RD (Li et al., 2010; Luo and Lan, 2010; Zhang and Xue, 2010; Bian et al., 2011; Guo and Shi, 2011; Li et al., 2011; Wang C. et al., 2011; Lin et al., 2013; Liang and Huang, 2014; Wang et al., 2014; Yang and Guan, 2014; Zhang, 2014; Zu et al., 2014; Mao, 2015; Zhang, 2015; Dong, 2019; Wang et al., 2019; Song et al., 2020; Lei et al., 2021; Liu, 2021). Due to the huge heterogeneity, we were unable to conduct a meta-analysis. Fourteen of the 20 studies showed that there was no statistical difference in the incidence of RD between TCHM and conventional therapy. Among the 14 trials, 10 studies showed that the incidence of RD was 100% in both groups (Li et al., 2010; Zhang and Xue, 2010; Bian et al., 2011; Guo and Shi, 2011; Lin et al., 2013; Zhang, 2014; Dong, 2019; Song et al., 2020; Lei et al., 2021; Liu, 2021), the remaining 4 studies showed that the incidence rate was about 80% in both groups (Wang et al., 2014; Yang and Guan, 2014; Zu et al., 2014; Mao, 2015). Six of the 20 studies have showed a difference between the two groups (Luo and Lan, 2010; Li et al., 2011; Wang J. et al., 2011; Liang and Huang, 2014; Zhang, 2015; Wang et al., 2019). Compared to conventional therapy, the incidence of RD is reduced by 10–90% in TCHM group. The specific results of each study are shown in Table 2. Subgroups classified by types of cancer, radiation dose, and dosage form of the TCHM could not explain the source of heterogeneity.

**TABLE 2:** Effect of value for incidence rate of radiodermatitis with TCHM.

Study	TCHM		Conventional therapy		Weight	Risk ratio
	Events	Total	Events	Total		M-H, random,95% CI
BianJL2011	60	60	60	60	5.70%	1.00 [0.97, 1.03]
DongQ2019	15	15	15	15	5.50%	1.00 [0.88, 1.13]
GuoYH2011	23	23	23	23	5.60%	1.00 [0.92, 1.09]
LeiL2021	38	38	38	38	5.70%	1.00 [0.95, 1.05]
LiangJ2014	8	79	73	73	2.50%	0.11 [0.06, 0.20]
LiJ2011	42	50	50	50	5.50%	0.84 [0.74, 0.95]
LiS2013	63	63	63	63	5.70%	1.00 [0.97, 1.03]
LiuXJ2021	19	19	18	18	5.60%	1.00 [0.90, 1.11]
LiXH2010	30	30	30	30	5.70%	1.00 [0.94, 1.07]
LuoAJ2010	11	50	35	50	2.90%	0.31 [0.18, 0.55]
MaoWP2015	16	20	17	20	4.60%	0.94 [0.71, 1.25]
SongFL2019	20	20	20	20	5.60%	1.00 [0.91, 1.10]
WangCZ2011	16	49	43	47	3.70%	0.36 [0.24, 0.54]
WangYC2019	31	90	61	90	4.40%	0.51 [0.37, 0.70]
WangYH2014	40	53	44	49	5.20%	0.84 [0.70, 1.01]
YangXY2014	31	56	49	56	4.80%	0.63 [0.49, 0.82]
ZhangJK2015	120	140	134	140	5.60%	0.90 [0.83, 0.97]
ZhangSS2014	36	36	36	36	5.70%	1.00 [0.95, 1.05]
ZhangWW2010	30	30	30	30	5.70%	1.00 [0.94, 1.07]
ZuGH2014	10	13	13	13	4.40%	0.78 [0.57, 1.07]

##### 3.4.2.2 Radiation Therapy Oncology Group Grading

**FIGURE 7:**
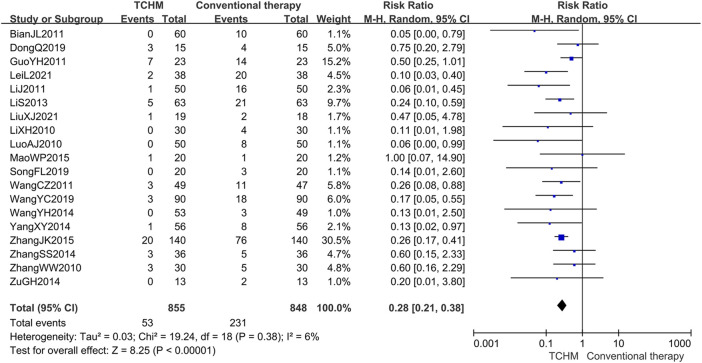
Forest plot and pooled risk ratios for association of RTOG grading criteria with TCHM and conventional therapy.

A total of 1703 patients in 19 studies reported RTOG grading (Li et al., 2010; Luo and Lan, 2010; Zhang and Xue, 2010; Bian et al., 2011; Guo and Shi, 2011; Li et al., 2011; Wang C. et al., 2011; Lin et al., 2013; Wang et al., 2014; Yang and Guan, 2014; Zhang, 2014; Zu et al., 2014; Mao, 2015; Zhang, 2015; Dong, 2019; Wang et al., 2019; Song et al., 2020; Lei et al., 2021; Liu, 2021). Meta-analysis showed that compared with conventional therapy, TCHM had an advantage in reducing RTOG grading (REM, RR = 0.28, 95%CI 0.21 to 0.38, 1703 participants, *I* ^*2*^ = 6%, *p* < 0.00001) (shown in Figure 7).

##### 3.4.2.3 Skin and Mucosa Recovery Time

Three studies including 358 patients reported skin and mucosa recovery time (Wang et al., 2014; Wang et al., 2019; Lei et al., 2021). Due to the huge heterogeneity, we were unable to conduct a meta-analysis. These studies showed that TCM topical medicine could shorten the recovery time of skin and mucosa compared with conventional education, and the MD values (95%CI) were respectively −5.53 (−6.63, −4.43) days; −4.64 (−5.18, −4.10) days; −19.40 (−21.79, −17.01) days.

##### 3.4.2.4 The Occurrence Time of Radiodermatitis

Two studies including 178 patients reported this outcome (Wang et al., 2014; Lei et al., 2021). Due to huge heterogeneity, we were unable to conduct a meta-analysis. One study showed that TCHM could delay the occurrence of radiation dermatitis for 4 days compared to conventional therapy. Another study showed that there was no difference in this outcome between groups.

##### 3.4.2.5 The Radiation Dose

No study mentioned this outcome.

##### 3.4.2.6 Quality of Life

Two studies including 77 patients reported quality of life (Mao, 2015; Lei et al., 2021). Due to the huge heterogeneity, we were unable to conduct a meta-analysis. One study showed that TCHM could improve patients’ quality of life compared with conventional therapy, while another study showed that there was no difference in this outcome between groups.

##### 3.4.2.7 Adverse Events

Six studies mentioned the adverse events (Wang et al., 2014; Mao, 2015; Zhang, 2015; Dong, 2019; Lei et al., 2021; Liu, 2021), all of them reported no adverse events.

### 3.5 Funnel Plot

**FIGURE 8:**
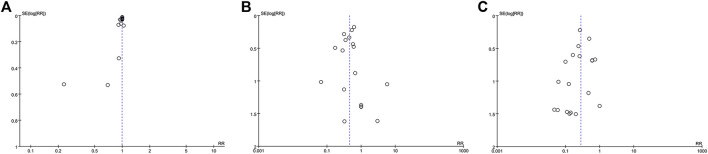
Funnel plot **(A)** TCHM compared to routine drugs with the incidence of radiation dermatitis reported in 22 trails **(B)** TCHM compared to routine drugs with RTOG grading criteria reported in 20 trails **(C)** TCHM compared to conventional therapy with RTOG grading criteria reported in 19 trails.

The funnel plot within data from 18 studies concerning TCHM compared to routine drugs with the incidence of radiation dermatitis/RTOG grading reduction showed potential asymmetry (Figures 8A,B). The funnel plot within data from 19 studies concerning TCHM compared to conventional therapy with RTOG Grading reduction showed potential asymmetry (Figure 8C). These may be caused by both of small study effect and publication bias.

### 3.6 Sensitivity Analysis

According to the protocol, sensitivity analysis was used to evaluate the influence of removing the high-risk study and switching the random/fixed-effect model on the results. There was only two high-risk trials in our study, the results showed that there was no difference after removing the high-risk trial from the meta-analysis (the result from original Figure 7 to RR = 0.27, 95%CI 0.20 to 0.36, *p* < 0.00001). After switching random/fixed-effect model, only one meta-analysis was changed (the result from original Figure 5 to RR = 0.97, 95%CI 0.94 to 0.99, *p* = 0.005). Since RR value is close to 1 and the huge heterogeneity in these trials, we still adopt the result from random-effect model.

### 3.7 Quality of Evidence

As shown in Table 3, when comparing TCHM to routine drugs, the quality of evidence was low for RTOG grading criteria, time of incidence of radiodermatitis, and very low for the incidence rate of radiodermatitis, recovery time of skin mucosa. As for the outcome of recovery time of skin mucosa and time of incidence of radiodermatitis, the publication bias of these endpoints was not used for GRADE evaluation since only 4 studies were included. As shown in Table 4, when comparing TCHM to conventional therapy, the quality of evidence was low for RTOG grading criteria.

**TABLE 3:** Summary of finding table of TCHM compared to routine drugs for radiodermatitis.

Summary of findings						
TCHM compared to routine drugs for radio dermatitis						
**Patient or population**: radiodermatitis						
**Setting**: outpatient department/inpatient department						
**Intervention**: topical medication of Chinese herbal medicine						
**Comparison**: routine drugs						
**Outcome N° of participants (studies)**	**Relative effect (95% CI)**	**Anticipated absolute effects (95% CI)**			**Certainty**	**What happens**
		**Without topical medication of Chinese herbal medicine**	**With topical medication of Chinese herbal medicine**	**Difference**		
Incidence rate of radiodermatitis N° of participants: 1,584 (18 RCTs)	**RR 0.99** (0.97–1.01)	92.8%	**91.9%** (90–93.7)	**0.9% fewer** (2.8 fewer to 0.9 more)	⊕○○○ VERY LOW^ab^ ^,^ ^c^	TCHM may reduce/have little to no effect on incidence rate of radiodermatitis but the evidence is very uncertain
RTOG (Radiation Therapy Oncology Group) Grading criteria assessed with: the ratio of 3 to 4grade patients to total patients N° of participants: 1,584 (18 RCTs)	**RR 0.46** (0.35–0.60)	29.0%	**13.3%** (10.0–17.4)	**15.6% fewer** (18.8 fewer to 11.6 fewer)	⊕⊕○○ LOW^a^ ^,^ ^c^	TCHM may reduce RTOG (Radiation Therapy Oncology Group) Grading criteria
Recovery time of skin mucosa assessed with: time N° of participants: 301 (4 RCTs)	-	The mean recovery time of skin mucosa was **24.76** days	-	MD **2.35** **days lower** (3.58 lower to 1.12 lower)	⊕○○○ VERY LOW^ab^ ^,^ ^d^	TCHM may reduce recovery time of skin mucosa
Time of incidence of radiodermatitis assessed with: time N° of participants: 334 (4 RCTs)	-	The mean time of incidence of radiodermatitis was **11.09** days	-	MD **2.36** **days higher** (1.74 higher to 2.98 higher)	⊕⊕○○ LOW^a^ ^,^ ^d^	TCHM may delay the time of incidence of radiodermatitis

*The risk in the intervention group (and its 95% confidence interval) is based on the assumed risk in the comparison group and the **relative effect** of the intervention (and its 95% CI). **CI:** Confidence interval; **RR:** Risk ratio; **MD:** Mean difference. **GRADE Working Group grades of evidence High certainty:** We are very confident that the true effect lies close to that of the estimate of the effect. **Moderate certainty:** We are moderately confident in the effect estimate: The true effect is likely to be close to the estimate of the effect, but there is a possibility that it is substantially different. **Low certainty:** Our confidence in the effect estimate is limited: The true effect may be substantially different from the estimate of the effect. **Very low certainty:** We have very little confidence in the effect estimate: The true effect is likely to be substantially different from the estimate of effect. Explanations:

a There were serious limitations of methodological quality among trials according to the risk of bias assessment.

b There was significant statistical heterogeneity among trials according to I.2.

c There were serious publication bias according to funnel plot.

d Too wide confidence interval.

**TABLE 4:** Summary of finding table of topical medication of Chinese herbal medicine compared to conventional therapy for radiodermatitis.

Summary of Findings						
TCHM compared to conventional therapy for radiodermatitis						
**Patient or population**: radiodermatitis						
**Setting**: outpatient department/Inpatient department						
**Intervention**: topical medication of Chinese herbal medicine						
**Comparison**: conventional therapy						
**Outcome № of participants (studies)**	**Relative effect (95% CI)**	**Anticipated absolute effects (95% CI)**			**Certainty**	**What happens**
		**Without topical medication of Chinese herbal medicine**	**With topical medication of Chinese herbal medicine**	**Difference**		
RTOG (Radiation Therapy Oncology Group) Grading criteria assessed with: the ratio of 3 to 4grade patients to total patients N° of participants: 1703 (19 RCTs)	**RR 0.28** (0.21–0.38)	27.2%	**7.6%** (5.7–10.4)	**19.6% fewer** (21.5 fewer to 16.9 fewer)	⊕⊕○○ LOW^a^ ^,^ ^b^	TCHM may reduce RTOG (Radiation Therapy Oncology Group) Grading criteria

*The risk in the intervention group (and its 95% confidence interval) is based on the assumed risk in the comparison group and the **relative effect** of the intervention (and its 95% CI). **CI:** Confidence interval; **RR:** Risk ratio. **GRADE Working Group grades of evidence**. **High certainty:** We are very confident that the true effect lies close to that of the estimate of the effect. **Moderate certainty:** We are moderately confident in the effect estimate: The true effect is likely to be close to the estimate of the effect, but there is a possibility that it is substantially different. **Low certainty:** Our confidence in the effect estimate is limited: The true effect may be substantially different from the estimate of the effect. **Very low certainty:** We have very little confidence in the effect estimate: The true effect is likely to be substantially different from the estimate of effect. Explanations:

a There were serious limitations of methodological quality among trials according to the risk of bias assessment.

b There were serious publication bias according to funnel plot.

## 4 Discussion

### 4.1 The Main Findings of the Review

A total of 38 studies were included in this study. Most of them were having some concerns about the risk of bias according to ROB 2.0 assessment. Very low-quality evidence was found that TCHM had no better effect on reducing the incidence rate of RD compared to routine drugs. However, low-quality evidence was found that TCHM may lower the RTOG grade of patients with RD (15.6% fewer than routine drugs) and delay the incidence of RD for about 2 days. Very low-quality evidence showed that TCHM may also cure the patients’ skin mucosa 2 days faster compared to routine drugs. When compared to conventional therapy, we got low-quality evidence that TCHM may reduce the RTOG grade (average 19.6% fewer than control). Insufficient evidence leads to inconclusive results on the safety of TCHM in preventing RD.

### 4.2 Advantages and the Limitations of the Review

Only the Chinese and English databases are searched, but TCHM is also used in some other countries, so the literature retrieval may be incomplete. As all included studies in this review reported acute RD, no studies concerning chronic RD as found, which may affect the reliability of the conclusions. Two studies reported missing data, and the missing data ratio was below 10%. We consider the impact of the missing data on the current conclusion may not be significant, so we did not use intention-to-treat analysis during the data synthesis.

Although several previously published systematic reviews are similar to our research topics, they are all different from this review. Ding et al. (2021) evaluated the therapeutic effect of TCHM for patients with RD, they focused on the cure rate and the cure time for RD patients treated by TCHM, but our review was aimed to evaluate the preventive effect of TCHM for patients with cancers who were about to accept radiation therapy. Hu and Hu (2019) included only six studies to evaluate the preventive effect of aloe gel for RD, which were concerned with all kinds of TCHM. Zheng (2021) did not target herbal medicine for preventing RD. Thus, this may be the first systematic review to assess the preventive effect of TCHM for RD.

Although we included 23 different prescriptions, the herbal medicine repetition rate in the prescriptions was 49.1% See Table 5 for specific patented formulations, botanical or chemical cited in the article. Meanwhile, the principle of all prescriptions is consistent, which is to clear heat-toxin. With the appropriate statistical analysis methods and the pre-designed subgroup analysis, we minimized the impact of clinical heterogeneity on the results and objectively evaluated the quality of evidence according to GRADE criteria. Although the quality of the evidence is low, it seems that TCHM can reduce the severity of RD, delay its occurrence, and accelerate the healing of skin lesions.

**TABLE 5:** Patented formulations, botanical or chemical, of the articles cited.

Study	Formulation	Source	Species, concentration	Quality control reported? (Y/N)	Chemical analysis reported? (Y/N)
DongQ2019	Mei-Bao’s scald plasters	Shantou Meibao Pharmaceutical co, LTD. (Guo Yao Zhun zi: Z20000004)	*Coptis chinensis Franch. [Ranunculaceae], Phellodendron amurense Rupr. [Rutaceae], Scutellaria baicalensis Georgi [Lamiaceae], Papaver somniferum L. [Papaveraceae]*	Y- Prepared according to The Pharmacopoeia of the People’s Republic of China	Y-TLC, HPLC
GuoYH2011					
NiuLY2013					
WangYC2019					
ZhangSS2014					
ZhangWW2010					
Fady BG2018		Julphar-Gulf Pharmaceutical Industries, UAE			
YangXY2014	Jingwan Hong Ointment	Tianjin Darentang pharmaceutical Jingwanhong Co. Ltd.	*Sanguisorba officinalis L. [Rosaceae], Angelica sinensis (Oliv.) Diels [Apiaceae], Prunus persica (L.) Batsch [Rosaceae], Lithospermum erythrorhizon Siebold & Zucc. [Boraginaceae], Lonicera japonica Thunb. [Caprifoliaceae], Rhus chinensis Mill. [Anacardiaceae], Angelica dahurica (Hoffm.) Benth. & Hook.f. ex Franch. & Sav. [Apiaceae], Calamus draco Willd. [Arecaceae], Dryobalanops aromatica C.F.Gaertn. [Dipterocarpaceae], Papaver somniferum L. [Papaveraceae], Rehmannia glutinosa (Gaertn.) DC. [Orobanchaceae], Coptis chinensis Franch. [Ranunculaceae]*	Y- Prepared according to The Pharmacopoeia of the People’s Republic of China	Y- HPLC
YuanHF2017	Chuangyang Ling	Armed Police Forces Hospital of Henan (Wu Wei Yao Zhun Zi: 60,105)	*Foeniculum vulgare Mill. [Apiaceae], Dryobalanops aromatica C.F.Gaertn. [Dipterocarpaceae], Pueraria montana* var. *lobata (Willd.) Maesen & S.M.Almeida ex Sanjappa & Predeep [Fabaceae], Angelica dahurica (Hoffm.) Benth. & Hook.f. ex Franch. & Sav. [Apiaceae]etc.*	Y - Quality controlled by Armed Police Forces Hospital of Henan	Y- Gas Chromatography (GC)
ZuGH2014	Fangshe Fanghu Ointment	Institute of Radiation Medicines, SDAMS (Patent No.201010607866)	*Angelica sinensis (Oliv.) Diels [Apiaceae] 30g, Dictamnus dasycarpus Turcz. [Rutaceae] 30g, Glycyrrhiza glabra L. [Fabaceae] 15g, Sophora flavescens Aiton [Fabaceae] 15g, Angelica dahurica (Hoffm.) Benth. & Hook.f. ex Franch. & Sav. [Apiaceae] 15g, Lithospermum erythrorhizon Siebold & Zucc. [Boraginaceae] 3g, Calamus draco Willd. [Arecaceae] 3g, Dryobalanops aromatica C.F.Gaertn. [Dipterocarpaceae] 3g*	Y - Prepared according to Pharmacopoeia of The People’s Republic of China	Y- HPLC
WangJ2016	Kuiyang Oil(a)	Dalian Municipal Central Hospital	*Lithospermum erythrorhizon Siebold & Zucc. [Boraginaceae], Carthamus tinctorius L. [Asteraceae], Rheum officinale Baill. [Polygonaceae], Angelica sinensis (Oliv.) Diels [Apiaceae], Astragalus mongholicus Bunge [Fabaceae] (All medicine are equal in weight)*	Y - Quality controlled by Dalian Municipal Central Hospital	Y- HPLC
YuZY2010		Nan Yang Tumour Hospital,, The First Affiliated Hospital of Guangdong Pharmaceutical University		Y - Quality controlled by Nan Yang Tumour Hospital &The First Affiliated Hospital of Guangdong Pharmaceutical University	
ZhaoRL2016		Yunnan *Cancer* Hospital		Y - Quality controlled by Yunnan *Cancer* Hospital	
WangXP2015	Kuiyang Oil(b)	China-Japan Friendship Hospital	*Lithospermum erythrorhizon Siebold & Zucc. [Boraginaceae], Carthamus tinctorius L. [Asteraceae], Rheum officinale Baill. [Polygonaceae], Paeonia lactiflora Pall. [Paeoniaceae], Astragalus mongholicus Bunge [Fabaceae] (All medicine are equal in weight)*	Y - Quality controlled by China-Japan Friendship Hospital	Y- HPLC
LiaoDZ2019	Huanglian Jiedu Liquid	Hospital (T.C.M) Affiliated to Southwest Medical University	*Coptis chinensis Franch. [Ranunculaceae], Scutellaria baicalensis Georgi [Lamiaceae], Phellodendron amurense Rupr. [Rutaceae], Lithospermum erythrorhizon Siebold & Zucc. [Boraginaceae], Dryobalanops aromatica C.F.Gaertn. [Dipterocarpaceae]*	Y - Quality controlled by Hospital (T.C.M) Affiliated to Southwest Medical University	Y-HPLC
WangYH2014	Huzhang gum bletilla film	The Pharmacy Departmengt of Daqing Oilfield General Hospital (Batch No.20101,027、20,110,602)	*Rheum officinale Baill. [Polygonaceae], Coptis chinensis Franch. [Ranunculaceae], Phellodendron amurense Rupr. [Rutaceae], Scutellaria baicalensis Georgi [Lamiaceae], Bletilla striata (Thunb.) Rchb.f. [Orchidaceae]*	Y - Quality controlled by Daqing Oilfield General Hospital	Y-TLC, HPLC
LiJH2013	Ruyi Jinhuang Powder	The People’s Hospital of Langfang City	*Curcuma longa L. [Zingiberaceae] 160g, Rheum officinale Baill. [Polygonaceae] 160g, Phellodendron amurense Rupr. [Rutaceae] 160g, Atractylodes lancea (Thunb.) DC. [Asteraceae] 64g, Magnolia officinalis Rehder & E.H.Wilson [Magnoliaceae] 64g, Citrus × aurantium L. [Rutaceae] 64g, Glycyrrhiza glabra L. [Fabaceae] 64g, Arisaema erubescens (Wall.) Schott [Araceae] 64g, Angelica dahurica (Hoffm.) Benth. & Hook.f. ex Franch. & Sav. [Apiaceae] 160g, Trichosanthes kirilowii Maxim. [Cucurbitaceae]320g*	Y - Prepared according to Pharmacopoeia of The People’s Republic of China	Y- HPLC
LiS2013	Tuhuang Lian Liqud	Guilin Hospital of Traditional Chinese Medicine (Gui Wei Yao Zhun Zi: Z03060028)	*Isodon serra (Maxim.) Kudô [Lamiaceae]*	Y - Quality controlled by Guilin Hospital of Traditional Chinese Medicine	Y-HPLC
WangHY2018	Aloe Liquid	Guangxi Medical University Affiliated Tumor & Oncology Medical College	*Aloe vera (L.) Burm.f. [Asphodelaceae]*	Y - Quality controlled by Guangxi Medical University Affiliated Tumor & Oncology Medical College	N
LiXH2010	Bingpian Huashi Powder	The Second People’s Hospital of Huaihua City	*Dryobalanops aromatica C.F.Gaertn. [Dipterocarpaceae]: Talci Pulvis = 1:2*	Y - Quality controlled by The Second People’s Hospital of Huihua City	N
LuoAJ2010				Y - Quality controlled by The Second People’s Hospital of Huaihua City	N
TangHJ2017	Fenghuang Liquid	Guilin Hospital of Chinese Traditional and Western Medicine	*Rheum officinale Baill. [Polygonaceae] 250g, Reynoutria japonica Houtt. [Polygonaceae] 250g*	Y - Quality controlled by Guilin Hospital of Chinese Traditional and Western Medicine	N
LiuXJ2021	Jiawei Simiao Yong’an Ointment	Beijing University of Chinese Medicine Third Affiliated Hospital	*Scrophularia ningpoensis Hemsl. [Scrophulariaceae] 90g, Lonicera japonica Thunb. [Caprifoliaceae] 90g, Angelica sinensis (Oliv.) Diels [Apiaceae] 60g, Astragalus mongholicus Bunge [Fabaceae] 30g, Lithospermum erythrorhizon Siebold & Zucc. [Boraginaceae] 30g, Forsythia suspensa (Thunb.) Vahl [Oleaceae] 30g, Taraxacum mongolicum Hand. -Mazz. [Asteraceae] 30g, Glycyrrhiza glabra L. [Fabaceae] 10g*	Y - Quality controlled by Beijing University of Chinese Medicine Third Affiliated Hospital	N
MaoWP2015				Y - Quality controlled by Beijing University of Chinese Medicine Third Affiliated Hospital	N
SongFL2019				Y - Quality controlled by Beijing University of Chinese Medicine Third Affiliated Hospital	N
YangWB2016				Y - Quality controlled by Beijing University of Chinese Medicine Third Affiliated Hospital	N
WangJG2011	Kangfu Xin Liquid	Huangzhou District People’s Hospital	*Nepeta tenuifolia Benth. [Lamiaceae], Forsythia suspensa (Thunb.) Vahl [Oleaceae], Paeonia lactiflora Pall. [Paeoniaceae], Gardenia jasminoides J.Ellis [Rubiaceae], Rehmannia glutinosa (Gaertn.) DC. [Orobanchaceae], Angelica dahurica (Hoffm.) Benth. & Hook.f. ex Franch. & Sav. [Apiaceae], Glycyrrhiza glabra L. [Fabaceae], Platycodon grandiflorus (Jacq.) A. DC. [Campanulaceae], Scutellaria baicalensis Georgi [Lamiaceae], Bupleurum Chinense DC. [Apiaceae], Phellodendron amurense Rupr. [Rutaceae], Mentha canadensis L. [Lamiaceae], Angelica sinensis (Oliv.) Diels [Apiaceae], Saposhnikovia divaricata (Turcz. ex Ledeb.) Schischk. [Apiaceae], Coptis chinensis Franch. [Ranunculaceae], Conioselinum anthriscoides ‘Chuanxiong’ [Apiaceae], Xanthium strumarium subsp. strumarium [Asteraceae], Bassia scoparia (L.) A.J.Scott [Amaranthaceae], Lithospermum erythrorhizon Siebold & Zucc. [Boraginaceae]etc.*	Y - Quality controlled by Huangzhou District People’s Hospital	N
LiJ2011	Liangxue Jiedu Ointment	The fourth hospital of Hebei Medical University	*Rheum officinale Baill. [Polygonaceae], Lithospermum erythrorhizon Siebold & Zucc. [Boraginaceae], Sanguisorba officinalis L. [Rosaceae], Aloe vera (L.) Burm.f. [Asphodelaceae], Isatis tinctoria L. [Brassicaceae], Cistanche deserticola Ma [Orobanchaceae], Taraxacum mongolicum Hand.-Mazz. [Asteraceae], Dryobalanops aromatica C.F.Gaertn. [Dipterocarpaceae]*	Y - Quality controlled by the fourth hospital of Hebei Medical University	N
WangCZ2011				Y - Quality controlled by the fourth hospital of Hebei Medical University	N
ZhangJK2015				Y - Quality controlled by the fourth hospital of Hebei Medical University	N
WuH2019	Qingshu You Ointment	Jiangsu Province Hospital of Chinese Medicine	*Scutellaria baicalensis Georgi [Lamiaceae] 30g, Lonicera japonica Thunb. [Caprifoliaceae] 30g, Sanguisorba officinalis L. [Rosaceae] 30g, Rehmannia glutinosa (Gaertn.) DC. [Orobanchaceae] 30g, Paeonia lactiflora Pall. [Paeoniaceae] 30g*	Y - Quality controlled by Jiangsu Province Hospital of Chinese Medicine	N
LiXJ2013	Sanhuang Ointment(a)	The Third People’s Hospital of Jinan (Patent No.CN101401866B)	*Scutellaria baicalensis Georgi [Lamiaceae], Coptis chinensis Franch. [Ranunculaceae], Phellodendron amurense Rupr. [Rutaceae], Salvia miltiorrhiza Bunge [Lamiaceae], Lithospermum erythrorhizon Siebold & Zucc. [Boraginaceae], Panax notoginseng (Burkill) F.H.Chen [Araliaceae], Dryobalanops aromatica C.F.Gaertn. [Dipterocarpaceae]*	Y - Quality controlled by The Third People’s Hospital of Jinan	N
XuY2014	Sanhuang Ointment(b)	Zhang Ye People’s Hospital Affiliated to He Xi University	*Scutellaria baicalensis Georgi [Lamiaceae], Phellodendron amurense Rupr. [Rutaceae], Coptis chinensis Franch. [Ranunculaceae]*	Y - Quality controlled by Zhang Ye People’s Hospital Affiliated to He Xi University	N
BianJL2011	Self-prescribed prescription(a)	The Third Hospital of Baoding City	*Angelica dahurica (Hoffm.) Benth. & Hook.f. ex Franch. & Sav. [Apiaceae], Lithospermum erythrorhizon Siebold & Zucc. [Boraginaceae], Angelica sinensis (Oliv.) Diels [Apiaceae], Calamus draco Willd. [Arecaceae], Boswellia carteri Birdw. [Burseraceae], Commiphora myrrha (T.Nees) Engl. [Burseraceae], Dryobalanops aromatica C.F.Gaertn. [Dipterocarpaceae]*	Y - Quality controlled by The Third Hospital of Baoding City	N
LiuFF2014	Self-prescribed prescription(b)	Weihai Central Hospital (Patent No. ZL2010106074509)	*Lithospermum erythrorhizon Siebold & Zucc. [Boraginaceae] 15g, Coptis chinensis Franch. [Ranunculaceae] 6g, Rheum officinale Baill. [Polygonaceae] 15g, Boswellia carteri Birdw. [Burseraceae] 12g, Commiphora myrrha (T.Nees) Engl. [Burseraceae] 12g*	Y - Quality controlled by Weihai Central Hospital	N
LeiL2021	Zibai Huangqi Ointment	Baotou *Cancer* Hospital	*Angelica sinensis (Oliv.) Diels [Apiaceae], Paeonia lactiflora Pall. [Paeoniaceae], Lithospermum erythrorhizon Siebold & Zucc. [Boraginaceae], Sanguisorba officinalis L. [Rosaceae], Astragalus mongholicus Bunge [Fabaceae], Bletilla striata (Thunb.) Rchb.f. [Orchidaceae]*	Y - Quality controlled by Baotou *Cancer* Hospital	N
LiangJ2014				Y - Quality controlled by Ruikang Hospital Affiliated to Guangxi University of Chinese Medicine	N
ZhaoSL2022	Qingre Yufu Ointment	Langfang People’s Hospital of Hebei Province	*Lonicera japonica Thunb. [Caprifoliaceae]30g, Scrophularia ningpoensis Hemsl. [Scrophulariaceae]30g, Astragalus mongholicus Bunge [Fabaceae]30g, Taraxacum mongolicum Hand. -Mazz. [Asteraceae]30g, Lithospermum erythrorhizon Siebold & Zucc. [Boraginaceae]30g, Forsythia suspensa (Thunb.) Vahl [Oleaceae]30g, Angelica sinensis (Oliv.) Diels [Apiaceae]20g, Dryobalanops aromatica C.F.Gaertn. [Dipterocarpaceae]10g*	Y - Quality controlled by Langfang People’s Hospital of Hebei Province	N

Italic values provided in Table 5 showed the Latin names and doses of the herbs used in the herbal prescriptions of each study.

### 4.3 Implications for Clinical Practice

This review suggests that topical herbal medicine can be used for the cancer patients who are about to accept radiotherapy to prevent severe RD and to accelerate the healing of skin lesions. The main treatment principle of the included trials is heat-clearing since, in TCM theory, radiation therapy is kind of an exogenous dry-heat toxin. Thus, *Lithospermum erythrorhizon Siebold & Zucc [Boraginaceae], Coptis chinensis Franch [Ranunculaceae], Phellodendron amurense Rupr [Rutaceae], Scutellaria baicalensis Georgi [Lamiaceae], Dryobalanops aromatica C.F.Gaertn [Dipterocarpaceae]* and other herbs with an effect on clearing heat-toxin can be used in self-made prescriptions during the treatment. The most frequently used herbal plant in this review is Mei-Bao’s scald plasters, and the most frequently used herb is *Lithospermum erythrorhizon Siebold & Zucc. [Boraginaceae]*. The possible optimal time for application of topical herbal medicine is 24–30 h before radiotherapy. In addition, the radiation dose included in this review was between 45 and 80 Gy.

### 4.4 Instructions for Future Research

Most parts of the studies included in this review may not be reported strictly in accordance with CONSORT reporting standards, so all results are rated as low or very low at the time of GRADE rating. In the future, researchers should conduct well-designed, high-quality clinical studies in accordance with CONSORT guidelines to verify the efficacy and safety of local use of TCHM in the prevention of radiation dermatitis. In future studies, we can consider the type of cancer as a variable to explore the dominant diseases. In addition, some studies in this review do not specify the specific dose of traditional Chinese medicine or the ratio between drugs, and the relationship between dose and efficacy is not clear, which may lead to differences in the results of different studies, and the composition of TCM compound prescription is often not clear. It limits the extrapolation of clinical research results. In future research, we should pay attention to the relationship between the dose and the efficacy of TCM to determine the best drug dose.

## 5 Conclusion

This study has shown that TCHM can reduce the severity of RD, delay its occurrence, and accelerate the healing of skin lesions. However, the evidence is low or very low due to the low quality of the included studies. Furthermore, the efficacy of topical medication of Chinese herbal medicine in the prevention of radiodermatitis still needs to be verified in future well-designed, high-quality clinical trials that adhere to CONSORT guidelines.

## Data Availability

The original contributions presented in the study are included in the article/Supplementary Material; further inquiries can be directed to the corresponding author.

## Abbreviations

TCHM, topical Chinese herbal medicine; RTOG, Radiation Therapy Oncology Group; RR, risk ratio; CI, confidence intervals; MD, mean difference; RCT, randomized controlled trials; RD, radiodermatitis; TCM, traditional Chinese medicine; CENTRAL, Cochrane Central Register of Controlled Trials; CNKI, the Chinese National Knowledge Infrastructure Databases; VIP, the Chongqing VIP Chinese Science and Technology Periodical Database; GRADE, Grading of Recommendations Assessment Development and Evaluation criteria; CONSORT, Consolidated Standards Of Reporting Trials; T, treatment; C, control; X, average; SD, standard deviation; BC, breast *cancer*; NPC, nasopharynx *cancer*; LCA, lung *cancer*; HCC, hepatocellular carcinoma; ESCA, esophageal carcinoma; GC, gastric *cancer*; TCS, thyroid carcinosarcoma; THCA, thyroid carcinoma; UCC, uterine cervix *cancer*; CRC, colorectal *cancer*.
