# Supplementary material for: Prevention of Radiodermatitis With Topical Chinese Herbal Medicine: A Systematic Review and Meta-Analysis
Source: Front Pharmacol. 2022 Jun 22;13:819733. doi: 10.3389/fphar.2022.819733 (PMC9257048; doi:10.3389/fphar.2022.819733)
Supplement: Supplementary file 2 [file DataSheet2.doc]

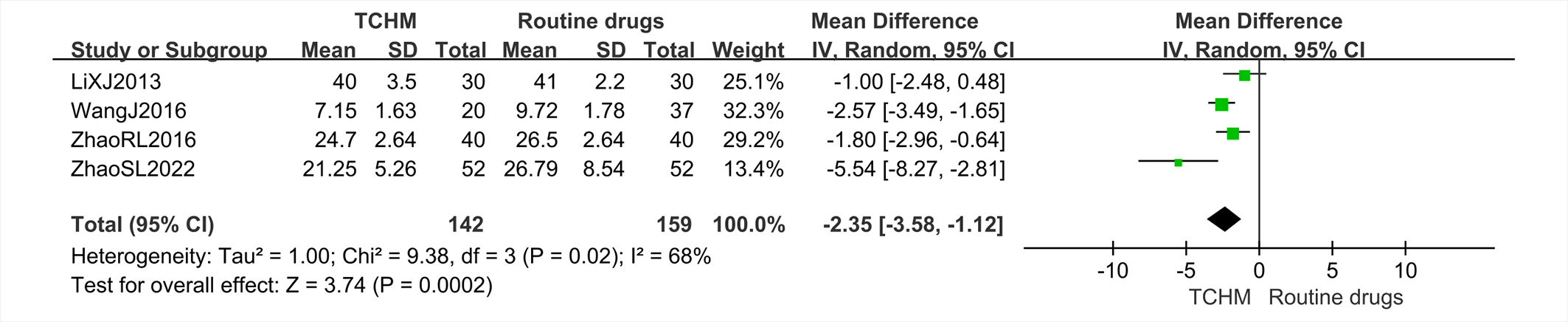


**FIGURE S1** │ Forest plot and pooled risk ratios for association of skin and mucosa recovery time with TCHM and Routine drugs.


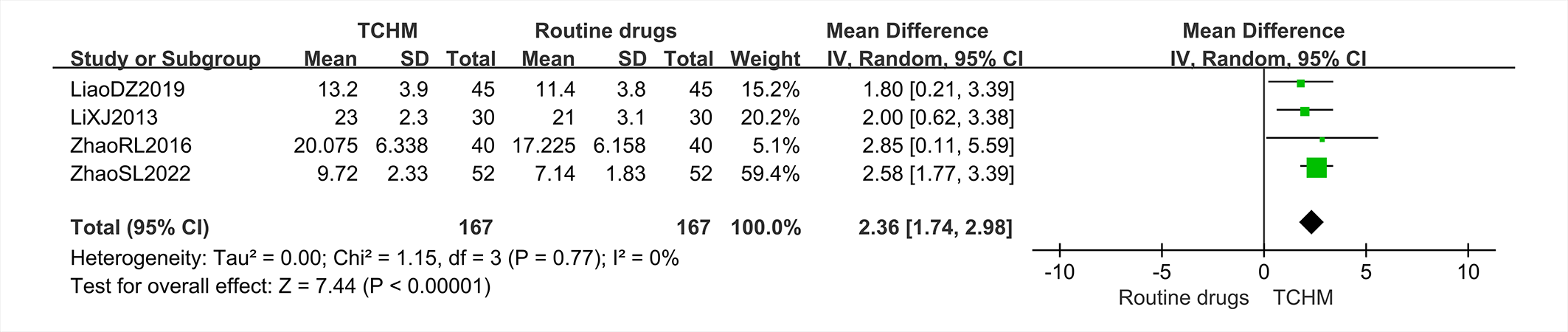


**FIGURE S2** │ Forest plot and pooled risk ratios for association of the occurrence time of radiation dermatitis with TCHM and Routine drugs.


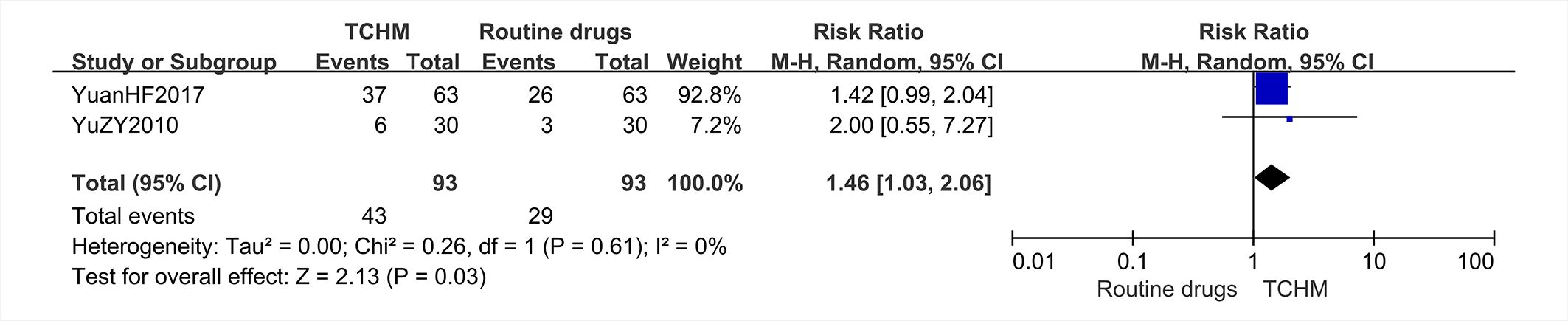


**FIGURE S3** │ Forest plot and pooled risk ratios for association of quality of life with TCHM and Routine drugs.

.

**Annexed table1. Prescription details form**

| **Study ID** | **TCHM** | **Principle of treatment** | **Sovereign medicinal** | **Prescription and dosege** |
| --- | --- | --- | --- | --- |
| **1.TCHM VS Conventional therapy** | | | | |
| LiXH2010 | Bingpian Huashi Powder | clearing heat-toxin, drying dampness and relieving pain, detumescence | *Dryobalanops aromatica C.F.Gaertn. [Dipterocarpaceae]* | *Dryobalanops aromatica C.F.Gaertn. [Dipterocarpaceae]:Talci Pulvis=1:2* |
| LuoAJ2010 |  |  |  |  |
| ZuGH2014 | Fangshe Fanghu Ointment | Drying dampness and relieving itching, clearing heat-toxin, healing up sore and promoting granulation | *Angelica sinensis (Oliv.) Diels [Apiaceae],Dictamnus dasycarpus Turcz. [Rutaceae],Lithospermum erythrorhizon Siebold & Zucc. [Boraginaceae]* | *Angelica sinensis (Oliv.) Diels [Apiaceae] 30g, Dictamnus dasycarpus Turcz. [Rutaceae] 30g, Glycyrrhiza glabra L. [Fabaceae] 15g, Sophora flavescens Aiton [Fabaceae] 15g, Angelica dahurica (Hoffm.) Benth. & Hook.f. ex Franch. & Sav. [Apiaceae] 15g, Lithospermum erythrorhizon Siebold & Zucc. [Boraginaceae] 3g, Calamus draco Willd. [Arecaceae] 3g, Dryobalanops aromatica C.F.Gaertn. [Dipterocarpaceae] 3g* |
| MaoWP2015 | Jiawei Simiao Yong'an Ointment | clearing heat-toxin, replenishing qi and nourishing yin, activating blood and promoting granulation | *Lonicera japonica Thunb. [Caprifoliaceae]* | *Scrophularia ningpoensis Hemsl. [Scrophulariaceae] 90g,* *Lonicera japonica Thunb. [Caprifoliaceae] 90g, Angelica sinensis (Oliv.) Diels [Apiaceae] 60g, Astragalus mongholicus Bunge [Fabaceae] 30g, Lithospermum erythrorhizon Siebold & Zucc. [Boraginaceae] 30g, Forsythia suspensa (Thunb.) Vahl [Oleaceae] 30g, Taraxacum mongolicum Hand.-Mazz. [Asteraceae] 30g, Glycyrrhiza glabra L. [Fabaceae] 10g* |
| LiuXJ2021 |  |  |  |  |
| SongFL2019 |  |  |  |  |
| YangXY2014 | Jingwan Hong Ointment | clearing heat-toxin, activating blood and detumescenc, eliminate putridity and promoting granulation | *Sanguisorba officinalis L. [Rosaceae],Angelica sinensis (Oliv.) Diels [Apiaceae],Prunus persica (L.) Batsch [Rosaceae]，Lithospermum erythrorhizon Siebold & Zucc. [Boraginaceae]，Lonicera japonica Thunb. [Caprifoliaceae]* | *Sanguisorba officinalis L. [Rosaceae], Angelica sinensis (Oliv.) Diels [Apiaceae], Prunus persica (L.) Batsch [Rosaceae], Lithospermum erythrorhizon Siebold & Zucc. [Boraginaceae], Lonicera japonica Thunb. [Caprifoliaceae], Rhus chinensis Mill. [Anacardiaceae], Angelica dahurica (Hoffm.) Benth. & Hook.f. ex Franch. & Sav. [Apiaceae], Calamus draco Willd. [Arecaceae], Dryobalanops aromatica C.F.Gaertn. [Dipterocarpaceae], Papaver somniferum L. [Papaveraceae],Rehmannia glutinosa (Gaertn.) DC. [Orobanchaceae], Coptis chinensis Franch. [Ranunculaceae]* |
| LiJ2011 | Liangxue Jiedu Ointment | Cooling blood and resoving toxin, clearing heat and promoting eruption, breaking and expelling blood stasis, activating blood and dredging collateral | *Rheum officinale Baill. [Polygonaceae],Lithospermum erythrorhizon Siebold & Zucc. [Boraginaceae],Sanguisorba officinalis L. [Rosaceae]* | *Rheum officinale Baill. [Polygonaceae], Lithospermum erythrorhizon Siebold & Zucc. [Boraginaceae], Sanguisorba officinalis L. [Rosaceae], Aloe vera (L.) Burm.f. [Asphodelaceae], Isatis tinctoria L. [Brassicaceae], Cistanche deserticola Ma [Orobanchaceae], Taraxacum mongolicum Hand.-Mazz. [Asteraceae], Dryobalanops aromatica C.F.Gaertn. [Dipterocarpaceae]* |
| WangCZ2011 |  |  |  |  |
| ZhangJK2015 |  |  |  |  |
| DongQ2019 | Mei-Bao's scald plasters | clearing heat-toxin, relieving pain, promoting granulation | *Coptis chinensis Franch. [Ranunculaceae],Phellodendron amurense Rupr. [Rutaceae] ,Scutellaria baicalensis Georgi [Lamiaceae]* | *Coptis chinensis Franch. [Ranunculaceae], Phellodendron amurense Rupr. [Rutaceae], Scutellaria baicalensis Georgi [Lamiaceae],, Papaver somniferum L. [Papaveraceae]* |
| GuoYH2011 |  |  |  |  |
| WangYC2019 |  |  |  |  |
| ZhangSS2014 |  |  |  |  |
| ZhangWW2010 |  |  |  |  |
| BianJL2011 | Self prescribed prescription(a) | clearing heat-toxin, relieving pain, promoting granulation | *Lithospermum erythrorhizon Siebold & Zucc. [Boraginaceae],Angelica sinensis (Oliv.) Diels [Apiaceae],Dryobalanops aromatica C.F.Gaertn. [Dipterocarpaceae]* | *Angelica dahurica (Hoffm.) Benth. & Hook.f. ex Franch. & Sav. [Apiaceae], Lithospermum erythrorhizon Siebold & Zucc. [Boraginaceae], Angelica sinensis (Oliv.) Diels [Apiaceae], Calamus draco Willd. [Arecaceae], Boswellia carteri Birdw. [Burseraceae], Commiphora myrrha (T.Nees) Engl. [Burseraceae], Dryobalanops aromatica C.F.Gaertn. [Dipterocarpaceae]* |
| WangYH2014 | Huzhang gum bletilla film | clearing heat-toxin, activating blood and resolving stasis, promoting granulation | *Rheum officinale Baill. [Polygonaceae],Bletilla striata (Thunb.) Rchb.f. [Orchidaceae]* | *Rheum officinale Baill. [Polygonaceae], Coptis chinensis Franch. [Ranunculaceae], Phellodendron amurense Rupr. [Rutaceae], Scutellaria baicalensis Georgi [Lamiaceae], Bletilla striata (Thunb.) Rchb.f. [Orchidaceae]* |
| LiS2013 | Tuhuang Lian Liqud | Clearing heat and drying dampness, purging fire and removing toxin | *Isodon serra (Maxim.) Kudô [Lamiaceae]* | *Isodon serra (Maxim.) Kudô [Lamiaceae]* |
| LeiL2021 | Zibai Huangqi Ointment | clearing heat-toxin, activating blood and resoving stasis, cooling blood, promoting granulation | *Lithospermum erythrorhizon Siebold & Zucc. [Boraginaceae],Bletilla striata (Thunb.) Rchb.f. [Orchidaceae],Astragalus mongholicus Bunge [Fabaceae]* | *Angelica sinensis (Oliv.) Diels [Apiaceae], Paeonia lactiflora Pall. [Paeoniaceae], Lithospermum erythrorhizon Siebold & Zucc. [Boraginaceae], Sanguisorba officinalis L. [Rosaceae], Astragalus mongholicus Bunge [Fabaceae], Bletilla striata (Thunb.) Rchb.f. [Orchidaceae]* |
| LiangJ2014 | Zicao Diyu Oil | clearing heat-toxin, stopping bleeding and cooling blood, activating blood and resoving stasis and dispering macule | *Lithospermum erythrorhizon Siebold & Zucc. [Boraginaceae],Sanguisorba officinalis L. [Rosaceae]* | *Lithospermum erythrorhizon Siebold & Zucc. [Boraginaceae], Sanguisorba officinalis L. [Rosaceae], Senecio scandens Buch.-Ham. ex D.Don [Asteraceae], Rheum officinale Baill. [Polygonaceae], Taraxacum mongolicum Hand.-Mazz. [Asteraceae]* |
| **2.TCHM VS Routine drugs** | | | | |
| WangHY2018 | Aloe Liquid | clearing heat-toxin, replenishing qi and nourishing yin | *Aloe vera (L.) Burm.f. [Asphodelaceae]* | *Aloe vera (L.) Burm.f. [Asphodelaceae]* |
| YuanHF2017 | Chuangyang Ling | clearing heat-toxin, replenishing qi and nourishing yin, activating blood and resoving stasis | *Pueraria montana var. lobata (Willd.) Maesen & S.M.Almeida ex Sanjappa & Predeep [Fabaceae]* | *Foeniculum vulgare Mill. [Apiaceae], Dryobalanops aromatica C.F.Gaertn. [Dipterocarpaceae], Pueraria montana var. lobata (Willd.) Maesen & S.M.Almeida ex Sanjappa & Predeep [Fabaceae], Angelica dahurica (Hoffm.) Benth. & Hook.f. ex Franch. & Sav. [Apiaceae], etc.* |
| TangHJ2017 | Fenghuang Liquid | clearing heat-toxin | *Rheum officinale Baill. [Polygonaceae]，Reynoutria japonica Houtt. [Polygonaceae]* | *Rheum officinale Baill. [Polygonaceae] 250g, Reynoutria japonica Houtt. [Polygonaceae] 250g,* |
| LiaoDZ2019 | Huanglian Jiedu Liquid | Clearing heat and drying dampness, removing toxin and detumescence, eliminate putridity and promoting granulation | *Coptis chinensis Franch. [Ranunculaceae],Scutellaria baicalensis Georgi [Lamiaceae],Phellodendron amurense Rupr. [Rutaceae]* | *Coptis chinensis Franch. [Ranunculaceae], Scutellaria baicalensis Georgi [Lamiaceae], Phellodendron amurense Rupr. [Rutaceae], Lithospermum erythrorhizon Siebold & Zucc. [Boraginaceae], Dryobalanops aromatica C.F.Gaertn. [Dipterocarpaceae]* |
| YangWB2016 | Jiawei Simiao Yong'an Ointment | clearing heat-toxin, replenishing qi and nourishing yin, activating blood and promoting granulation | *Lonicera japonica Thunb. [Caprifoliaceae]* | *Lonicera japonica Thunb. [Caprifoliaceae] 30ｇ, Scrophularia ningpoensis Hemsl. [Scrophulariaceae] 30ｇ, Angelica sinensis (Oliv.) Diels [Apiaceae] 20ｇ, Glycyrrhiza glabra L. [Fabaceae] 10ｇ, Astragalus mongholicus Bunge [Fabaceae] 30ｇ, Forsythia suspensa (Thunb.) Vahl [Oleaceae] 30ｇ, Taraxacum mongolicum Hand.-Mazz. [Asteraceae] 30ｇ, Lithospermum erythrorhizon Siebold & Zucc. [Boraginaceae] 30ｇ, Dryobalanops aromatica C.F.Gaertn. [Dipterocarpaceae] 10ｇ* |
| WangJG et al2011 | Kangfu Xin Liquid | clearing heat-toxin, activating blood and resoving stasis, strengthening vital qi and promoting granulation | *Coptis chinensis Franch. [Ranunculaceae],Scutellaria baicalensis Georgi [Lamiaceae],Phellodendron amurense Rupr. [Rutaceae]，Lithospermum erythrorhizon Siebold & Zucc. [Boraginaceae]* | *Nepeta tenuifolia Benth. [Lamiaceae], Forsythia suspensa (Thunb.) Vahl [Oleaceae], Paeonia lactiflora Pall. [Paeoniaceae], Gardenia jasminoides J.Ellis [Rubiaceae], Rehmannia glutinosa (Gaertn.) DC. [Orobanchaceae], Angelica dahurica (Hoffm.) Benth. & Hook.f. ex Franch. & Sav. [Apiaceae], Glycyrrhiza glabra L. [Fabaceae], Platycodon grandiflorus (Jacq.) A.DC. [Campanulaceae], Scutellaria baicalensis Georgi [Lamiaceae], Bupleurum chinense DC. [Apiaceae], Phellodendron amurense Rupr. [Rutaceae], Mentha canadensis L. [Lamiaceae], Angelica sinensis (Oliv.) Diels [Apiaceae], Saposhnikovia divaricata (Turcz. ex Ledeb.) Schischk. [Apiaceae], Coptis chinensis Franch. [Ranunculaceae], Conioselinum anthriscoides 'Chuanxiong' [Apiaceae], Xanthium strumarium subsp. strumarium [Asteraceae], Bassia scoparia (L.) A.J.Scott [Amaranthaceae], Lithospermum erythrorhizon Siebold & Zucc. [Boraginaceae], etc.* |
| WangJ2016 | Kuiyang Oil(a) | clearing heat-toxin, replenishing qi and nourishing yin, activating blood and resoving stasis | *Astragalus mongholicus Bunge [Fabaceae]* | *Lithospermum erythrorhizon Siebold & Zucc. [Boraginaceae], Carthamus tinctorius L. [Asteraceae], Rheum officinale Baill. [Polygonaceae], Angelica sinensis (Oliv.) Diels [Apiaceae], Astragalus mongholicus Bunge [Fabaceae] (All medicine are equal in weight)* |
| YuZY2010 |  |  |  |  |
| ZhaoRL2016 |  |  |  |  |
| WangXP2015 | Kuiyang Oil(b) |  |  | *Lithospermum erythrorhizon Siebold & Zucc. [Boraginaceae], Carthamus tinctorius L. [Asteraceae], Rheum officinale Baill. [Polygonaceae], Paeonia lactiflora Pall. [Paeoniaceae], Astragalus mongholicus Bunge [Fabaceae] (All medicine are equal in weight)* |
| NiuLY2013 | Mei-Bao's scald plasters | clearing heat-toxin, relieving pain, promoting granulation | *Coptis chinensis Franch. [Ranunculaceae],Scutellaria baicalensis Georgi [Lamiaceae],Phellodendron amurense Rupr. [Rutaceae]* | *Phellodendron amurense Rupr. [Rutaceae], Scutellaria baicalensis Georgi [Lamiaceae], Coptis chinensis Franch. [Ranunculaceae],, Papaver somniferum L. [Papaveraceae]* |
| Fady BG2018 |  |  |  |  |
| LiJH2013 | Ruyi Jinhuang Powder | clearing heat-toxin, detumescence relieving pain | *Rheum officinale Baill. [Polygonaceae] ,Phellodendron amurense Rupr. [Rutaceae]* | *Curcuma longa L. [Zingiberaceae] 160g, Rheum officinale Baill. [Polygonaceae] 160g, Phellodendron amurense Rupr. [Rutaceae] 160g, Atractylodes lancea (Thunb.) DC. [Asteraceae] 64g, Magnolia officinalis Rehder & E.H.Wilson [Magnoliaceae] 64g, Citrus × aurantium L. [Rutaceae] 64g, Glycyrrhiza glabra L. [Fabaceae] 64g, Arisaema erubescens (Wall.) Schott [Araceae] 64g, Angelica dahurica (Hoffm.) Benth. & Hook.f. ex Franch. & Sav. [Apiaceae] 160g, Trichosanthes kirilowii Maxim. [Cucurbitaceae]320g* |
| LiXJ2013 | Sanhuang Ointment(a) | clearing heat-toxin, detumescence and dissipating binds | *Coptis chinensis Franch. [Ranunculaceae],Scutellaria baicalensis Georgi [Lamiaceae],Phellodendron amurense Rupr. [Rutaceae]* | *Scutellaria baicalensis Georgi [Lamiaceae], Coptis chinensis Franch. [Ranunculaceae], Phellodendron amurense Rupr. [Rutaceae], Salvia miltiorrhiza Bunge [Lamiaceae], Lithospermum erythrorhizon Siebold & Zucc. [Boraginaceae], Panax notoginseng (Burkill) F.H.Chen [Araliaceae], Dryobalanops aromatica C.F.Gaertn. [Dipterocarpaceae]* |
| XuY et al2014 | Sanhuang Ointment(b) | Clearing heat and drying dampness, cooling blood and removing toxin, stopping bleeding and activating blood | *Coptis chinensis Franch. [Ranunculaceae],Scutellaria baicalensis Georgi [Lamiaceae],Phellodendron amurense Rupr. [Rutaceae]* | *Scutellaria baicalensis Georgi [Lamiaceae], Phellodendron amurense Rupr. [Rutaceae], Coptis chinensis Franch. [Ranunculaceae]* |
| LiuFF2014 | Self prescribed prescription(b) | Clearing heat and cooling blood, resolving stasis and toxin, relieving pain and promoting granulation | *Lithospermum erythrorhizon Siebold & Zucc. [Boraginaceae]* | *Lithospermum erythrorhizon Siebold & Zucc. [Boraginaceae] 15g,, Coptis chinensis Franch. [Ranunculaceae] 6g, Rheum officinale Baill. [Polygonaceae] 15g, Boswellia carteri Birdw. [Burseraceae] 12g, Commiphora myrrha (T.Nees) Engl. [Burseraceae] 12g* |
| WuH2019 | Qingshu You Ointment | clearing heat-toxin, activating blood and resoving stasis | *Lonicera japonica Thunb. [Caprifoliaceae]* | *Scutellaria baicalensis Georgi [Lamiaceae] 30g, Lonicera japonica Thunb. [Caprifoliaceae] 30g, Sanguisorba officinalis L. [Rosaceae] 30g, Rehmannia glutinosa (Gaertn.) DC. [Orobanchaceae] 30g, Paeonia lactiflora Pall. [Paeoniaceae] 30g* |
| ZhaoSL2022 | Qingre Yufu Ointment | clearing heat-toxin, activating blood and detumescence, resoving stasis and relieving pain | *Lonicera japonica Thunb. [Caprifoliaceae]* | *Lonicera japonica Thunb. [Caprifoliaceae]30g, Scrophularia ningpoensis Hemsl. [Scrophulariaceae]30g, Astragalus mongholicus Bunge [Fabaceae]30g, Taraxacum mongolicum Hand.-Mazz. [Asteraceae]30g, Lithospermum erythrorhizon Siebold & Zucc. [Boraginaceae]30g, Forsythia suspensa (Thunb.) Vahl [Oleaceae]30g, Angelica sinensis (Oliv.) Diels [Apiaceae]20g, Dryobalanops aromatica C.F.Gaertn. [Dipterocarpaceae]10g* |

**[Attached](../../../../C:/Users/lenovo/AppData/Local/youdao/dict/Application/8.9.9.0/resultui/html/index.html#/javascript:;) table2. Effect of estimates of prevention of radiodermatitis with TCHM in 38 included trials**

| **Trials** | **Intervention/Comparison** | **Effect estimates(95%CI)** | **Pvalue** |
| --- | --- | --- | --- |
| **1.Incidence rate of radiodermatitis** | | | |
| 1.1 TCHM vs. Routine drugs | | | |
| Fady BG2018 | Mei-Bao's scald plasters vs. Trolamine Cream | RR 0.94 [0.89, 1.00] |  |
| LiaoDZ2019 | Huanglian Jiedu Liquid vs. Recombinant Human Epidermal Growth Factor Derivative For External Use,Liquid | RR 1.00 [0.96, 1.04] |  |
| LiJH2013 | Ruyi Jinhuang Powder vs. Recombinant Human Epidermal Growth Factor Derivative For External Use,Liquid | RR 1.00 [0.95, 1.05] |  |
| LiuFF2014 | Self prescribed prescription(b) vs. Superoxide Dismutase,liquid | RR 0.69 [0.24, 1.97] |  |
| LiXJ2013 | Sanhuang Ointment(a) vs. Trolamine Cream | RR 0.92 [0.48, 1.74] |  |
| NiuLY2013 | Mei-Bao's scald plasters vs. Trolamine Cream | RR 1.00 [0.94, 1.06] |  |
| TangHJ2017 | Fenghuang Liquid vs. Trolamine Cream | RR 0.23 [0.08, 0.65] |  |
| WangHY2018 | Aloe Liquid vs. Trolamine Cream | RR 0.97 [0.86, 1.10] |  |
| WangJ2016 | Kuiyang Oil(a) vs. Trolamine Cream | RR 1.00 [0.97, 1.03] |  |
| WangJG2011 | Kangfu Xin Liquid vs. Mucopolysaccharide Polysulfate Cream | RR 1.00 [0.97, 1.03] |  |
| WangXP2015 | Kuiyang Oil(b) vs. Trolamine Cream | RR 1.00 [0.95, 1.05] |  |
| WuH2019 | Qingshu You Ointment vs. Vaseline Ointment | RR 1.00 [0.97, 1.03] |  |
| XuY2014 | Sanhuang Ointment(b) vs. Magnesium Sulfate Ointment | RR 1.00 [0.95, 1.05] |  |
| YangWB2016 | Jiawei Simiao Yong'an Ointment vs.Trolamine Cream | RR 1.04 [0.89, 1.21] |  |
| YuanHF2017 | Chuangyang Ling vs. Compound Dexamethasone Acetate | RR 0.91 [0.79, 1.05] |  |
| YuZY2010 | Kuiyang Oil(a) vs. Trolamine Cream | RR 1.00 [0.94, 1.07] |  |
| ZhaoRL2016 | Kuiyang Oil(a) vs. Hydrocortisone Cream | RR 1.00 [0.95, 1.05] |  |
| ZhaoSL2022 | Qingre Yufu Ointment vs. Trolamine Cream | RR 0.96 [0.90, 1.03] |  |
| **Subtotoal(REM, I^2^=54%)** |  | RR 0.99 [0.97, 1.01] | **0.43** |
| 1.2 TCHM vs. Conventional therapy | | | |
| BianJL2011 | Self prescribed prescription(a) | RR 1.00 [0.97, 1.03] |  |
| DongQ2019 | Mei-Bao's scald plasters | RR 1.00 [0.88, 1.13] |  |
| GuoYH2011 | Mei-Bao's scald plasters | RR 1.00 [0.92, 1.09] |  |
| LeiL2021 | Zibai Huangqi Ointment | RR 1.00 [0.95, 1.05] |  |
| LiangJ2014 | Zicao Diyu Oil | RR 0.11 [0.06, 0.20] |  |
| LiJ2011 | Liangxue Jiedu Ointment | RR 0.84 [0.74, 0.95] |  |
| LiS2013 | Tuhuang Lian Liqud | RR 1.00 [0.97, 1.03] |  |
| LiuXJ2021 | Jiawei Simiao Yong'an Ointment | RR 1.00 [0.90, 1.11] |  |
| LiXH2010 | Bingpian Huashi Powder | RR 1.00 [0.94, 1.07] |  |
| LuoAJ2010 | Bingpian Huashi Powder | RR 0.31 [0.18, 0.55] |  |
| MaoWP2015 | Jiawei Simiao Yong'an Ointment | RR 0.94 [0.71, 1.25] |  |
| SongFL2019 | Jiawei Simiao Yong'an Ointment | RR 1.00 [0.91, 1.10] |  |
| WangCZ2011 | Liangxue Jiedu Ointment | RR 0.36 [0.24, 0.54] |  |
| WangYC2019 | Mei-Bao's scald plasters | RR 0.51 [0.37, 0.70] |  |
| WangYH2014 | Huzhang gum bletilla film | RR 0.84 [0.70, 1.01] |  |
| YangXY2014 | Jingwan Hong Ointment | RR 0.63 [0.49, 0.82] |  |
| ZhangJK2015 | Liangxue Jiedu Ointment | RR 0.90 [0.83, 0.97] |  |
| ZhangSS2014 | Mei-Bao's scald plasters | RR 1.00 [0.95, 1.05] |  |
| ZhangWW2010 | Mei-Bao's scald plasters | RR 1.00 [0.94, 1.07] |  |
| ZuGH2014 | Fangshe Fanghu Ointment | RR 0.78 [0.57, 1.07] |  |
| **Subtotoal(REM,I^2^=98%)** |  | **RR** 0.80 [0.70, 0.92] | **0.001** |
| **2. RTOG Grading criteria** | | | |
| 2.1 TCHM vs. Routine drugs | | | |
| Fady BG2018 | Mei-Bao's scald plasters vs. Trolamine Cream | RR 0.61 [0.24, 1.56] |  |
| LiaoDZ2019 | Huanglian Jiedu Liquid vs. Recombinant Human Epidermal Growth Factor Derivative For External Use,Liquid | RR 0.62 [0.43, 0.88] |  |
| LiJH2013 | Ruyi Jinhuang Powder vs. Recombinant Human Epidermal Growth Factor Derivative For External Use,Liquid | RR 1.00 [0.06, 15.44] |  |
| LiuFF2014 | Self prescribed prescription(b) vs. Superoxide Dismutase,liquid | RR 0.32 [0.01, 7.68] |  |
| LiXJ2013 | Sanhuang Ointment(a) vs. Trolamine Cream | RR 3.00 [0.13, 70.83] |  |
| TangHJ2017 | Fenghuang Liquid vs. Trolamine Cream | RR 1.00 [0.07, 14.55] |  |
| NiuLY2013 | Mei-Bao's scald plasters vs. Trolamine Cream | RR 5.81 [0.74, 45.54] |  |
| WangHY2018 | Aloe Liquid vs. Trolamine Cream | RR 0.67 [0.12, 3.75] |  |
| WangJ2016 | Kuiyang oil(a) vs. Trolamine Cream | RR 0.54 [0.35, 0.84] |  |
| WangJG2011 | Kangfu Xin Liquid vs. Mucopolysaccharide Polysulfate Cream | RR 0.07 [0.01, 0.49] |  |
| WangXP2015 | Kuiyang Oil(b) vs. Trolamine Cream | RR 0.32 [0.03, 2.90] |  |
| WuH2019 | Qingshu You Ointment vs. Vaseline Ointment | RR 0.29 [0.10, 0.82] |  |
| XuY2014 | Sanhuang Ointment(b) vs. Magnesium Sulfate Ointment | RR 0.35 [0.17, 0.73] |  |
| YangWB2016 | Jiawei Simiao Yong'an Ointment vs.Trolamine Cream | RR 1.00 [0.07, 15.26] |  |
| YuanHF2017 | Chuangyang Ling vs. Compound Dexamethasone Acetate | RR 0.58 [0.25, 1.38] |  |
| YuZY2010 | Kuiyang Oil(a) vs. Trolamine Cream | RR 0.44 [0.23, 0.86] |  |
| ZhaoRL2016 | Kuiyang Oil(a) vs. Hydrocortisone Cream | RR 0.17 [0.07, 0.46] |  |
| ZhaoSL2022 | Qingre Yufu Ointment vs. Trolamine Cream | RR 0.31 [0.18, 0.55] |  |
| **Subtotoal(FEM,I^2^=27%)** |  | **RR**  0.46 [0.35, 0.60] | **<0.00001** |
| 2.2 TCHM vs. Conventional therapy | | | |
| BianJL2011 | Self prescribed prescription(a) | RR 0.05 [0.00, 0.79] |  |
| DongQ2019 | Mei-Bao's scald plasters | RR 0.75 [0.20, 2.79] |  |
| GuoYH2011 | Mei-Bao's scald plasters | RR 0.50 [0.25, 1.01] |  |
| LeiL2021 | Zibai Huangqi Ointment | RR 0.10 [0.03, 0.40] |  |
| LiJ2011 | Liangxue Jiedu Ointment | RR 0.06 [0.01, 0.45] |  |
| LiS2013 | Tuhuang Lian Liqud | RR 0.24 [0.10, 0.59] |  |
| LiuXJ2021 | Jiawei Simiao Yong'an Ointment | RR 0.47 [0.05, 4.78] |  |
| LiXH2010 | Bingpian Huashi Powder | RR 0.11 [0.01, 1.98] |  |
| LuoAJ2010 | Bingpian Huashi Powder | RR 0.06 [0.00, 0.99] |  |
| MaoWP2015 | Jiawei Simiao Yong'an Ointment | RR 1.00 [0.07, 14.90] |  |
| SongFL2019 | Jiawei Simiao Yong'an Ointment | RR 0.14 [0.01, 2.60] |  |
| WangCZ2011 | Liangxue Jiedu Ointment | RR 0.26 [0.08, 0.88] |  |
| WangYC2019 | Mei-Bao's scald plasters | RR 0.17 [0.05, 0.55] |  |
| WangYH2014 | Huzhang gum bletilla film | RR 0.13 [0.01, 2.50] |  |
| YangXY2014 | Jingwan Hong Ointment | RR 0.13 [0.02, 0.97] |  |
| ZhangJK2015 | Liangxue Jiedu Ointment | RR 0.26 [0.17, 0.41] |  |
| ZhangSS2014 | Mei-Bao's scald plasters | RR 0.60 [0.15, 2.33] |  |
| ZhangWW2010 | Mei-Bao's scald plasters | RR 0.60 [0.16, 2.29] |  |
| ZuGH2014 | Fangshe Fanghu Ointment | RR 0.20 [0.01, 3.80] |  |
| **Subtotoal(REM,I^2^=6%)** |  | **RR** 0.20 [0.01, 3.80] | **<0.0001** |
| **3. Recovery time of skin mucosa** | | | |
| 3.1 TCHM vs. Routine drugs | | | |
| LiXJ2013 | Sanhuang Ointment(a) vs. Trolamine Cream | MD -1.00 [-2.48, 0.48] |  |
| WangJ2016 | Kuiyang Oil(a) vs. Trolamine Cream | MD -2.57 [-3.49, -1.65] |  |
| ZhaoRL2016 | Kuiyang Oil(a) vs. Hydrocortisone Cream | MD -1.80 [-2.96, -0.64] |  |
| ZhaoSL2022 | Qingre Yufu Ointment vs. Trolamine Cream | MD -5.54 [-8.27, -2.81] |  |
| **Subtotoal(REM,I^2^=68%)** |  | **MD -2.35 [-3.58, -1.12]** | **0.0002** |
| 3.2 TCHM vs. Adjuvant therapy | | | |
| LeiL2021 | Zibai Huangqi Ointment | MD -5.53[-6.63,-4.43] |  |
| WangYC2019 | Mei-Bao's scald plasters | MD -4.64[-5.18,-4.10] |  |
| WangYH2014 | Huzhang gum bletilla film | MD -19.40[-21.79,-17.01] |  |
| **Subtotoal(REM,I^2^=99%)** |  | **MD -5.39[-5.87,-4.92]** | **<0.00001** |
| **4. Time of incidence of radiodermatitis** | | | |
| 4.1 TCHM vs. Routine drugs | | | |
| LiaoDZ2019 | Huanglian Jiedu Liquid vs. Recombinant Human Epidermal Growth Factor Derivative For External Use,Liquid | MD 1.80 [0.21, 3.39] |  |
| LiXJ2013 | Sanhuang Ointment(a) vs. Trolamine Cream | MD 2.00 [0.62, 3.38] |  |
| ZhaoRL2016 | Kuiyang Oil(a) vs. Hydrocortisone Cream | MD 2.85 [0.11, 5.59] |  |
| ZhaoSL2022 | Qingre Yufu Ointment vs. Trolamine Cream | MD 2.58 [1.77, 3.39] |  |
| **Subtotoal(REM,I^2^=0%)** |  | **MD 2.36 [1.74, 2.98]** | **<0.00001** |
| 4.2 TCHM vs. Conventional therapy | | | |
| LeiL2021 | Zibai Huangqi Ointment | MD 4.00[2.12,5.88] |  |
| WangYH2014 | Huzhang gum bletilla film | MD -2.70[-3.94,-1.46] |  |
| **Subtotoal(REM,I^2^=97%)** |  | **MD 0.61[-5.95,7.18]** | **0.86** |
