# Supplementary material for: Prevention of Radiodermatitis With Topical Chinese Herbal Medicine: A Systematic Review and Meta-Analysis
Source: Front Pharmacol. 2022 Jun 22;13:819733. doi: 10.3389/fphar.2022.819733 (PMC9257048; doi:10.3389/fphar.2022.819733)
Supplement: Supplementary file 3 [file DataSheet1.docx]

**Supplementary material 1. Detailed search strategy**

**Supplementary Table A: Search Strategy Used in PubMed 2022/4/19**

| No. | Search items | Items found |
| --- | --- | --- |
| #1 | (Radiodermatitis[MeSH Terms]) OR (Radiodermatitides[MeSH Terms]) OR (Radiation-Induced Dermatitis[MeSH Terms]) OR (Radiation-Induced Dermatitides[MeSH Terms]) OR (Dermatitis, Radiation-Induced[MeSH Terms]) OR (Dermatitides, Radiation-Induced[MeSH Terms]) OR (Radiation Recall Dermatitis[MeSH Terms]) OR (Radiation Recall Dermatitides[MeSH Terms]) OR (Dermatitides, Radiation Recall[MeSH Terms]) OR (Dermatitis, Radiation Recall[MeSH Terms]) OR (Dermatitis, Radiation Recall[Title/Abstract]) OR (Dermatitides, Radiation Recall[Title/Abstract]) OR (Radiation Recall Dermatitides[Title/Abstract]) OR (\Radiation Recall Dermatitis[Title/Abstract]) OR (Dermatitides, Radiation-Induced[Title/Abstract]) OR (Dermatitis, Radiation-Induced[Title/Abstract]) OR (Radiation-Induced Dermatitides[Title/Abstract]) OR (Radiation-Induced Dermatitis[Title/Abstract]) OR (Radiodermatitides[Title/Abstract]) OR (Radiodermatitis[Title/Abstract]) | 3019 |
| #2 | (Medicine, Herbal[MeSH Terms]) OR (Traditional Chinese Medicine[MeSH Terms]) OR (Traditional Medicine, Chinese[MeSH Terms]) OR (Chinese Drugs, Plant[MeSH Terms]) OR (Chinese Herbal Drugs[MeSH Terms]) OR (Herbal Drugs, Chinese[MeSH Terms]) OR (Plant Extracts, Chinese[MeSH Terms]) OR (Chinese Plant Extracts[MeSH Terms]) OR (Extracts, Chinese Plant[MeSH Terms]) OR (Medicine, Herbal[Title/Abstract]) OR (Traditional Chinese Medicine[Title/Abstract]) OR (Traditional Medicine, Chinese[Title/Abstract]) OR (Chinese Drugs, Plant[Title/Abstract]) OR (Chinese Herbal Drugs[Title/Abstract]) OR (Herbal Drugs, Chinese[Title/Abstract]) OR (Plant Extracts, Chinese[Title/Abstract]) OR (Chinese Plant Extracts[Title/Abstract]) OR (Extracts, Chinese Plant[Title/Abstract]) | 81098 |
| #3 | #1 AND #2 | 10 |
| #4 | #1 AND #2 Filter:2010-2021 | 8 |

**Supplementary Table B: Search Strategy Used in CoChrane 2022/4/19**

| No. | Search items | Items found |
| --- | --- | --- |
| #1 | (Radiodermatitis): MESH | 336 |
| #2 | (Dermatitis, Radiation Induced): MESH | 287 |
| #3 | (Radiation Induced Dermatitis): MESH | 287 |
| #4 | (Radiation Induced-Dermatitis): MESH | 107 |
| #5 | (Radiation Induced-Dermatitides): MESH | 0 |
| #6 | (Dermatitides, Radiation-Induced): MESH | 0 |
| #7 | (Dermatitis, Radiation-Induced): MESH | 171 |
| #8 | (Radiation Recall Dermatitis): MESH | 6 |
| #9 | (Radiation Recall Dermatitis Dermatitides): MESH | 0 |
| #10 | (Dermatitides, Radiation Recall): MESH | 0 |
| #11 | #1 OR #2 OR #3 OR #4 OR #5 OR #6 OR #7 OR #8 #9 OR #10 | 539 |
| #12 | (Chinese Drug, Plant): MESH | 772 |
| #13 | (Herbal Drugs, Chinese): MESH | 4366 |
| #14 | (Chinese Plant Extracts): MESH | 341 |
| #15 | (Extracts, Chinese Plant): MESH | 341 |
| #16 | (Plant Extracts, Chinese): MESH | 341 |
| #17 | (Plant Extract, Chinese): MESH | 343 |
| #18 | (Chinese Medicine): MESH | 33149 |
| #19 | (Chinese herb): MESH | 793 |
| #20 | (Herbal medication): MESH | 1229 |
| #21 | #12 OR #13 OR #14 OR #15 OR #16 OR #17 OR #18 | 34015 |
| #22 | #11 AND #21  with Cochrane Library publication date from Jan 1,2010 to April 19,2022, in Trials. | 4 |

**Supplementary C: Search Strategy Used in Proquest 2022/4/19**



Items found: 3

(mainsubject.Exact("chinese medicine") OR mainsubject.Exact("herbal medicine") OR ti(Medicine, Herbal) OR ti(Traditional Chinese Medicine) OR ti(Traditional Medicine, Chinese) OR ti(Chinese drug, Plant) OR ti(Chinese Herbal drug) OR ti(Herbal drug, Chinese) OR ti(Herbal drug, Chinese) OR ti(Plant Extract, Chinese) OR ti(herb medication) OR ti(herbal medicine) OR ti(chinese medicine)) AND (mainsubject.Exact("radiodermatitis") OR ti(Radiodermatitis) OR ti(Radiodermatitises) OR ti(Radiation-Induced Dermatitis) OR ti(Radiation-Induced dermatitises) OR ti(Dermatitis, Radiation-Induced) OR ti(dermatitises, Radiation-Induced) OR ti(Radiation Recall Dermatitis) OR ti(Radiation Recall dermatitises) OR ti(dermatitises, Radiation Recall) OR ti(Dermatitis, Radiation Recall))

**Supplementary D: Search Strategy Used in Springer link 2022/4/19**



Items found: 32

("Chinese medicine" OR "herbal medicine" OR Medicine, Herbal OR "Traditional Chinese Medicine") AND ("radiodermatitis" OR "Radiation-Induced Dermatitis" OR "Radiation-Induced Dermatitides" OR "Dermatitis, Radiation-Induced" OR "Dermatitides, Radiation-Induced" OR "Radiation Recall Dermatitis" OR "Radiation Recall Dermatitides" OR "Dermatitides, Radiation Recall" OR "Dermatitis, Radiation Recall" OR "Dermatitis, Radiation Recall")

**Search Strategy Used in CNKI 2022/4/19**

Items found: 177

SU=('放射性皮损'+'放射性皮炎'+'放射性黏膜炎'+'放射后皮损'+'放射后皮炎'+'放射后黏膜炎') AND SU=('中医'+'中药'+'复方'+'中成药'+'汤药'+'中药敷料'+'中药合剂'+'中药外治'+'中药外治'+'中药外洗'+'膏'+'散'+'中药喷雾') AND FT=('随机')

**Supplementary Table E: Search Strategy Used in WangFang 2022/4/19**

Items found: 329

主题:(放射性皮损+放射性皮炎+放射性黏膜炎+放射后皮损+放射后黏膜炎+放射后皮炎)*主题:(中医+中药+复方+中成药+汤药 +中药敷料+中药合剂+中药外治+中药外用+中药外洗+膏+散+中药喷雾)*主题:(随机)

**Supplementary F: Search Strategy Used in VIP 2022/4/19**

Items found: 5

M=(放射性皮损 OR 放射性皮炎 OR 放射性黏膜炎 OR 放射后皮损 OR 放射后皮炎 OR 放射后黏膜炎) AND M=（中医 OR 中药 OR 复方 OR 中成药 OR 汤药 OR 中药敷料 OR 中药合剂 OR 中药外治 OR 中药外用 OR 中药外洗 OR 膏 OR 散 OR 中药喷雾) AND M=（随机）

**Supplementary G: Search Strategy Used in SCOPUS 2022/4/24**

Items found: 59

ALL ( ( radiodermatitis ) OR ( radiodermatitides ) OR ( "Radiation-Induced Dermatitis" ) OR ( "Radiation-Induced Dermatitides" ) OR ( "Dermatitis, Radiation-Induced" ) OR ( "Dermatitides, Radiation-Induced" ) OR ( "Radiation Recall Dermatitis" ) OR ( "Radiation Recall Dermatitides" ) OR ( "Dermatitides, Radiation Recall" ) OR ( "Dermatitis, Radiation Recall" ) OR ( "Dermatitis, Radiation Recall" ) ) AND ( ( "Medicine, Herbal" ) OR ( "Traditional Chinese Medicine" ) OR ( "Traditional Medicine, Chinese" ) OR ( "Chinese Drugs, Plant" ) OR ( "Chinese Herbal Drugs" ) OR ( "Herbal Drugs, Chinese" ) OR ( "Plant Extracts, Chinese" ) OR ( "Chinese Plant Extracts" ) OR ( "Extracts, Chinese Plant" ) )

**Supplementary H: Search Strategy Used in PsycINFO 2022/4/24**

Items found: 0

"radiodermatitis" OR "Radiation-Induced Dermatitis" OR "Radiation-Induced Dermatitides" OR "Radiation Recall Dermatitis" OR "Radiation Recall Dermatitides" AND "Chinese medicine" OR "herbal medicine" OR “Medicine, Herbal” OR "Traditional Chinese Medicine"

**Supplementary I: Search Strategy Used in ASSIA 2022/4/24**

Items found: 0

"radiodermatitis" OR "Radiation-Induced Dermatitis" OR "Radiation-Induced Dermatitides" OR "Radiation Recall Dermatitis" OR "Radiation Recall Dermatitides" AND "Chinese medicine" OR "herbal medicine" OR “Medicine, Herbal” OR "Traditional Chinese Medicine"

**Supplementary J: Search Strategy Used in PQDT 2022/4/24**

Items found: 0

"radiodermatitis" OR "Radiation-Induced Dermatitis" OR "Radiation-Induced Dermatitides" OR "Radiation Recall Dermatitis" OR "Radiation Recall Dermatitides" AND "Chinese medicine" OR "herbal medicine" OR “Medicine, Herbal” OR "Traditional Chinese Medicine"
